# Beneficial impacts of physical activity on heart rate variability: A systematic review and meta-analysis

**DOI:** 10.1371/journal.pone.0299793

**Published:** 2024-04-05

**Authors:** Ouahiba El-Malahi, Darya Mohajeri, Raluca Mincu, Alexander Bäuerle, Korbinian Rothenaicher, Ramtin Knuschke, Christos Rammos, Tienush Rassaf, Julia Lortz

**Affiliations:** 1 Department of Cardiology and Vascular Medicine, West-German Heart and Vascular Center Essen, University of Duisburg-Essen, Essen, Germany; 2 Clinic for Psychosomatic Medicine and Psychotherapy, LVR-University Hospital Essen, University of Duisburg-Essen, Essen, Germany; University of the Witwatersrand, SOUTH AFRICA

## Abstract

**Background:**

Cardiovascular diseases (CVD) are the leading causes of morbidity and mortality. Heart rate variability (HRV) represents the modulatory capacity of the autonomous nervous system and influences mortality. By surveying this meta-analysis, we investigated the impact of physical activity on HRV.

**Methods:**

Databases, online journal libraries and clinical trial registries were searched for publications of randomized controlled and non-randomized controlled trials concerning adults with coronary artery disease (CAD)/ischemic heart disease (IHD), congestive heart failure (CHF), peripheral arterial disease (PAD) or after acute coronary syndrome (ACS) joining an intervention group with physical activity or a control group with usual care or no intervention. Extracted time-domain and frequency-domain parameter of HRV were analyzed in a meta-analysis using a random effect model. Subgroup analyses concerning intervention type, study design and type of heart disease and sensitivity analysis were performed.

**Results:**

Significant results were obtained for RR-Interval (p = 0.05) and standard deviation of Normal-to-Normal intervals (SDNN) (p = 0.01) for short-term assessment and for the ratio of low-frequency power (LF) to high-frequency power (HF) (p = 0.05) for 24-hour assessment. Subgroup analyses also resulted significant: root-mean-square difference of successive normal R-R intervals (RMSSD) (p = 0.01), SDNN (p = 0.02) and HF (p < 0.01) concerning CHF.

**Conclusion:**

We were able to demonstrate the positive impact of physical activity on HRV, especially in patients with CHF. Cardiac rehabilitation exercise programs need to be individualized to identify the most beneficial method of training for improving the prognosis of patients with CVD.

## Background

Cardiovascular diseases (CVD) remain the leading cause of morbidity and mortality worldwide despite continuous advances in risk management and stratification [1]. There is growing evidence that exercise-based cardiac rehabilitation programs reduce morbidity and mortality in patients with CVD [2–5], since cardiorespiratory fitness assessed by heart rate (HR)-derived indices and parasympathetic nervous system activity are considered independent predictors of mortality [6–8]. As a major relevant HR-based component, heart rate variability (HRV) and its derivatives are applied as non-invasive and reliable parameters for the indirect measurement of parasympathetic activity. HRV is defined as the variation in time between two consecutive heartbeats and depends on continuous autonomic modulation [9]. A higher HRV indicates a higher level of modulatory capacity in the parasympathetic nervous system, whereas a lower HRV suggests a greater dominance of the sympathetic nervous system. Studies have also shown an association between lower HRV and an increased risk of mortality [10,11]. In patients with CVD, decreased HRV is also associated with major adverse cardiovascular events such as stroke or sudden cardiac death [12,13].

Relevant time-domain derivatives of HRV include the RR-Interval, the root-mean-square difference of successive normal R-R intervals (RMSSD) and the standard deviation of Normal-to-Normal intervals (SDNN). These time-domain derivatives typically decrease with age or as a result of psychological or physiological disorders, such as stress disorders or CVD [14–16]. The frequency-domain analysis includes different frequency bands such as high-frequency power (HF; 0.15–0.40 Hz) or low-frequency power (LF; 0.04–0.15 Hz) as well as the LF/HF ratio [14]. Among various parameters, parasympathetic activity is reflected by the frequency-domain parameter HF. A reduction in HF is frequently linked to mental reactions such as anxiety or stress, as well as various cardiac diseases. Opinions diverge regarding the frequency-domain parameter LF: It is generally used to indicate sympathetic nervous system activity. Recent studies propose that the frequency-domain parameter LF may be influenced by parasympathetic activity alone or by both parasympathetic and sympathetic activity [14,17,18]. The LF/HF ratio is often considered to represent the balance in the autonomic nervous system. In theory, a low LF/HF ratio may indicate dominance of the parasympathetic nervous system, while a high LF/HF ratio may suggest dominance of the sympathetic nervous system. However, this perspective is debated due to varying interpretations of LF and the complexities of interactions between the parasympathetic and sympathetic nervous systems [14,18].

Exercise-based cardiac rehabilitation programs showed to be beneficial for most patients increasing the function of the autonomic nervous system [19,20]. Predefined training programs might not suit each patient’s individual responsiveness that varies depending on the prevalent CVD. Therefore, the primary objective of this study was to determine whether physical training improves HR-derived indices to a greater extent depending on the underlying disease.

## Methods

This systematic literature review and meta-analysis was performed in compliance with the "Preferred Reporting of Items for Systematic Reviews and Meta-analyses (PRISMA)" guidelines and is registered on the “International Prospective Register of Systematic Reviews (PROSPERO)”, registration number CRD42022364185 [21]. Neither ethical approval nor written consent of the participants were required due to the fact that all analyses were conducted based on already published data.

### Search strategy

The databases PubMed, Embase and Cochrane Library were systematically searched for literature without any language restrictions using free text and/or Medical Subject Headings (MeSH) or Emtree. Completed and ongoing studies were also looked for in the clinical trial registries clinicaltrials.gov, German Clinical Trials Register (DRKS), International Clinical Trials Registry Platform (ICTRP) and International Standard Randomised Controlled Trial Number registry (ISRCTN registry). Appropriate publications were further researched in the online library of AHA/ASA Journals, The American Journal of Cardiology and International Journal of Cardiology by using free text.

We defined the following keywords: “heart rate variability“, “coronary artery disease“, “chronic ischemic heart disease“, “acute coronary syndrome“, “heart failure“, “peripheral arterial disease“, “cardiac rehabilitation“, “training“, “exercise“, “program”and “physical activity“. Combinations of several keywords were created using the operators “AND” as well as “OR”. The phrase search was used for keywords consisting of more than one word to maintain the exact string. MeSH terms were created using the MeSH database of PubMed. The created free text search term was converted for Embase and Cochrane Library using the Polyglot Search Translator [22]. MeSH terms were replaced for Embase, if necessary, by terms from Emtree in the converted search term after a manual matching between the MeSH terms and Emtree. The detailed search strategy can be found in S1 Table, Supplemental Material.

The following filter functions were used to specify the search:

- Pubmed: “Full text” (category: “Text availability”) and “Humans” (category: “Species”)
- Embase: “Controlled Clinical Trial” and “Randomized Controlled Trial” (category: “Evidence Based Medicine)
- ClinicalTrials.gov: “Interventional Studies (Clinical Trials)” (category: “Study type” using the advanced search mode)
- DRKS: “Interventional” (category: “Study type”)
- all journals: “Research Article” (category: “Article Type”).

### Selection process

All identified publications were imported into the literature management software EndNote (Version 20.4.1 for Windows, Clarivate Analytics). Detected duplicates were initially removed. Remaining publications were systematically screened for eligibility according to title and abstract and then decided on regarding full text screening, which was then performed in a second step. At least one reason for exclusion of a publication was documented during the screening processes in EndNote. Studies that had been included by researching a clinical trial registry underwent a further screening process step. A Google search was conducted using the clinical trial registry number to identify reported results of each study. In cases without any matching study report, results were requested by sending an e-mail to the given contact person. The request date was documented in EndNote.

Following criteria had to be met for inclusion: (i) adults, older than 18 years old with congestive heart failure (CHF), peripheral arterial disease (PAD), coronary artery disease (CAD)/ischemic heart disease (IHD) or after acute coronary syndrome (ACS); (ii) intervention group performing any physical activity (i.e. resistance training, aerobic training); (iii) control group with usual care (i.e. usual medical care, conservative medical care, phone calls for study adherence, ordinary acitivities) or no intervention; (iv) at least one time-domain or frequency-domain parameter of HRV reported as outcome; (v) study design had to be randomized controlled trial (RCT) or non-RCT.

Studies without accessible abstract or full text as well as studies that met one or more of the following criteria were excluded: (i) atrial fibrillation in all patients; (ii) yoga, pure inspiratory muscle training or biofeedback training as intervention type; (iii) studies without a control group; (iv) case-control studies, letters, reviews, meta-analyses and animal studies.

### Data extraction

The data extraction process consisted of two main parts. Firstly, data extraction for studies without accessible or published results that were collected from a clinical trial registry. For studies without access to the results, following information were recorded in a Microsoft Excel data sheet: Study name, study identification number, methods, participants, intervention, outcome, starting date and contact information. E-Mail requests or other relevant notes were recorded in the same data form in an additional column. The second part referred to data extraction regarding studies with accessible and published results, that were surveyed from databases and online journal libraries. For these, the following data were extracted in another Microsoft Excel data form: Name of the first author, year of publication, study identification number, general study information (study design, method of randomization, blinding, intention-to-treat-analysis), population size (at study start, end of study and for HRV-analysis), demographic data (mean age, age range and gender distribution), investigated heart disease and other reported diseases, inclusion and exclusion criteria of each study, applied intervention (type, duration, cycles), investigated parameter of HRV as outcome (assessment and analysis method for time-domain and/or frequency-domain parameter as well as used frequency range), number and reasons for drop-outs, results at baseline and after intervention period, reported p-values and conclusion of the author.

### Risk of bias assessment

In order to assess the risk of bias for RCTs the Risk-of-Bias-Tool 2 was used. An adapted version of the Risk-of-Bias-Tool 2 was applied for RCTs with cross-over design [23]. Non-RCTs were evaluated by “Risk-Of-Bias In Non-Randomized Studies–of Interventions” [24].

### Data synthesis and calculation

Baseline and post-interventional values were summarized for the time-domain parameters RR-Interval, SDNN, RMSSD and the frequency domain measurements for ultra-low frequency bands (ULF), very-low frequency bands (VLF), total power (TP), LF, LF in normalized units (nLF), HF, HF in normalized units (nHF) and LF/HF [14]. This was done for the intervention group as well as for the control group of each included study for meta-analysis in Microsoft Excel. All values were expressed as mean values with their standard deviation (SD). One included study investigated two intervention groups and one control group [25]. In order to avoid a loss of information by excluding one or both of these intervention groups from meta-analysis, the values of both intervention groups were converted to one intervention group by using Microsoft Excel for the calculation steps with regard to the standardized formulas of the Cochrane Handbook, Chapter 6.5.2.10, Table 6.5a [26].

To avoid a mixture of logarithmic data and non-logarithmic data in meta-analysis, logarithmically transformed values of outcome data were converted to raw data in Microsoft Excel according to one of the presented formula [27]. Published logarithmic values that could not be transformed to raw data because of either invalid calculation results or very high SD values compared to the mean values were not included in meta-analysis.

### Meta-analysis

Meta-analysis was conducted with IBM SPSS Statistics (Version 29.0.0.0 for Windows, International Business Machines Corporation) and was separated in short-term (less than 30 minutes recordings) and 24-hour assessment of HRV ensuring comparability. The meta-analysis included analysis of RR-Interval, SDNN and RMSSD for time-domain and TP, LF, HF, nHF and LF/HF for frequency-domain parameters. All analyses were performed using post-interventional values of both groups: the intervention and the control group.

A random effect model was used, and the standard mean difference (SMD) was chosen as an effect size. Heterogeneity was expressed using I^2^ statistics. Studies without published pre-interventional and post-interventional values but with published values of change were not included in meta-analysis. The resulted effect sizes of LF/HF were multiplied with the factor -1 to maintain in all forest plots the same order: Effect sizes within the right side of the axis favour the intervention and within the left side of the axis the control group.

A subgroup analysis was performed related to intervention type, study design and type of heart disease for different parameters using IBM SPSS Statistics as well. Additionally, the effect size “mean difference” was analyzed in a sensitivity analysis summarizing at least two studies with the same unit for each parameter. All subgroup analyses and the sensitivity analysis were conducted using the values RR-Interval, RMSSD, SDNN, LF and HF of short-term assessment. The 24-hour assessment was only used in relation to the sensitivity analysis of “mean difference”.

## Results

### Study selection and characteristics

**Fig 1:**
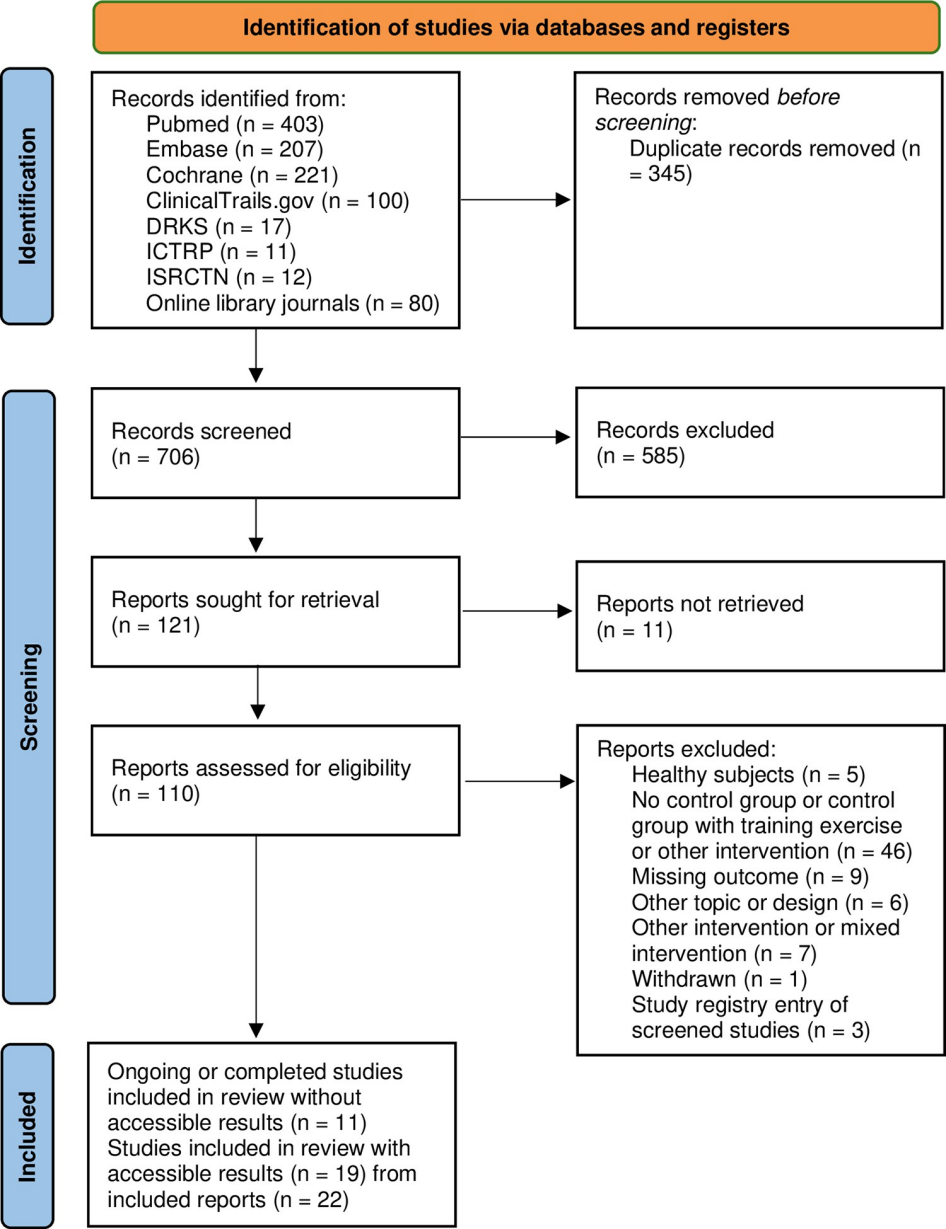
PRISMA flow chart illustrating steps and details of the identification and selection process for the systematic review.

The systematic literature research resulted in 706 publications after removing duplicates. In the next step, 33 publications were included after a full text screening. Of these, 11 publications were identified as completed or ongoing studies without accessible or published data and were separately presented in S2 Table, Supplemental Material. From the remaining publications, 19 studies were extracted from 22 included study reports. Details of this process are shown in the PRISMA flow chart [21], Fig 1 and the characteristics of these included studies are presented in Table 1. The majority of our included studies showed random assignment of the participants to an intervention or a control group [25,28–43]. The assessment of HRV parameters was conducted with a 24-hour electrocardiogram (ECG) recording in five studies [28,30,36–38], with a 20-hour ECG recording in one study [42], with short-term ECG recording in 10 studies [25,31,33–35,39,41,43–45] and lastly, three studies used the short-term HR recording method [29,32,40]. Mean age of all participants over all included studies with accessible results ranged from 52 ± 8 up to 72 ± 5 years [28,42]. The longest intervention period was 12 months compared to the shortest intervention period that showed to be six weeks [37,39].

**Table 1:** Characteristics of the 19 included studies.

Author	Year	Design	Disease	Physical activity	Period	HRV assessment	Sample size		Mean age	Female
Ståhle et al. [28]	1999	RCT	myocardial infarction, unstable angina pectoris	interval training	3 months	24-hour ECG	Intervention Control	29* 36*	71 ± 4 72 ± 5	7 8
Fiogbé et al. [29]	2018	RCT	coronary artery disease	water aerobic exercise training	16 weeks	Short-term HR	Intervention Control	14 12	60 ± 4 59 ± 3	0 0
Adamopoulos et al. [30]	1992	RCT	congestive heart failure	bicycle exercise training	8 weeks	24-hour ECG	Intervention Control			
Duru et al. [31]	2000	RCT	myocardial infarction, new-onset heart failure	outdoor walking and stationary cycling training	8 weeks	Short-term ECG	Intervention Control	12 13	56 ± 5 55 ± 7	0 0
Oliveira et al. [32]	2014	RCT	myocardial infarction	aerobic exercise training	8 weeks	Short-term HR	Intervention Control	47 45	55 ± 11 59 ± 11	7 8
Murad et al. [33]	2012	RCT	congestive heart failure	aerobic exercise training	16 weeks	Short-term ECG	Intervention Control	31 35	68 ± 5 70 ± 6	20 22
Ricca-Mallada et al. [34]	2012	RCT	congestive heart failure	aerobic and resistance exercise training	24 weeks	Short-term ECG	Intervention Control	10 10	59 ± 8 57 ± 8	2 2
Ricca-Mallada et al. [35]	2016	RCT	congestive heart failure	aerobic exercise training	24 weeks	Short-term ECG	Intervention Control	16 18	57 ± 10 56 ± 9	3 4
Munk et al. [36]	2009	RCT	coronary artery disease, angina pectoris	high intensity interval training	6 months	24-hour ECG	Intervention Control	20 18	58 ± 10 60 ± 9	3 3
Lai et al. [44]	2011	Non-RCT	coronary artery disease	walking exercise	8 weeks	Short-term ECG	Intervention Control	16 16	64 ± 6 67 ± 5	16 16
Sandercock et al. [25]	2007	RCT	peripheral arterial disease	walking exercise	12 weeks	Short-term ECG	Intervention I Intervention II Control	13 15 15	66 ± 8 62 ± 14 67 ± 6	4 3 5
Bilińska et al. [37]	2013	RCT	coronary artery disease	bicycle exercise training	6 weeks	24-hour ECG	Intervention Control	50 50	57 ± 6 56 ± 6	0 0
Piotrowicz et al. [38]	2014	RCT	congestive heart failure	walking exercise	8 weeks	24-hour ECG	Intervention Control	36 15	53 ± 10 61 ± 13	5 0
Leicht et al. [39]	2011	RCT	peripheral arterial disease	walking exercise	12 months	Short-term ECG	Intervention Control	8 9	68 ± 6 65 ± 9	4 4
Tamburus et al. [40]	2015	RCT	coronary artery disease	individualized interval training	16 weeks	Short-term HR	Intervention Control	12 10	56 ± 7 60 ± 6	0 0
Chang et al. [45]	2008	Non-RCT	coronary artery disease	T‘ai Chi training	9 months	Short-term ECG	Intervention Control	22 39	58 ± 11 66 ± 10	2 12
Selig et al. [41]	2004	RCT	congestive heart failure	resistance exercise training	3 months	Short-term ECG	Intervention Control	19* 20*	65 ± 13 64 ± 9	4 2
Kiilavuori et al. [42]	1995	RCT	congestive heart failure	bicycle exercise training	3 months	20-hour ECG	Intervention Control	8 12	52 ± 8 52 ± 10	0 1
Brenner et al. [43]	2020	RCT	peripheral arterial disease	walking exercise	12 weeks	Short-term ECG	Intervention Control	18 15	69 ± 7 64 ± 8	6 6

RCT = randomized-controlled trials; ECG = electrocardiogram; HR = heart rate; *Number of analyzed participants for all or some parameters of HRV different.

### Risk of bias

**Fig 2:**
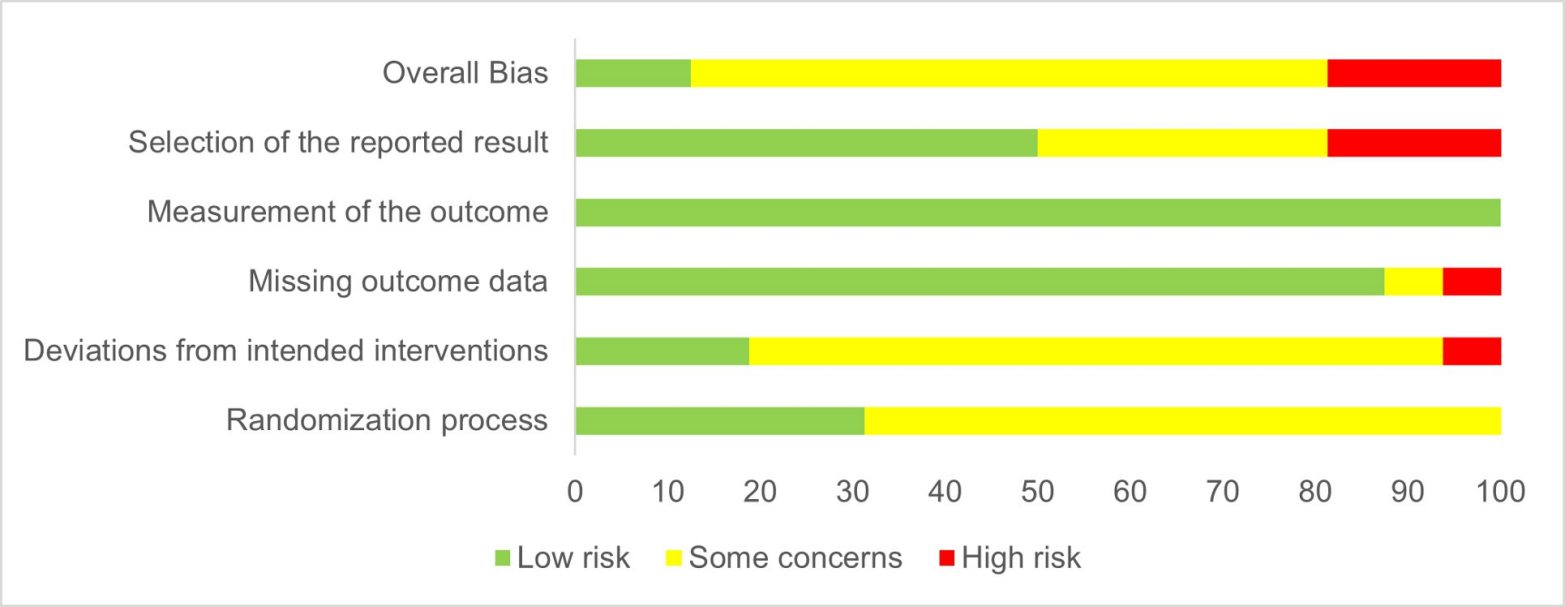
Risk of bias graph showing results of quality assessment for included RCTs without cross-over design concerning randomization process, deviations, missing outcome data, outcome measurement, selection of reported results and the resulting overall bias.

All included studies listed in Table 1 were assessed regarding the risk of bias. The resulting assessment of all RCTs is shown in Fig 2. Two studies were rated to an overall low risk of bias [25,29]. In terms of randomization process, no study was judged with a high risk of bias. One study rated to a high risk of bias [37] and one study was classified with some concerns [38] as a consequence of missing outcome data. In summary, all studies were evaluated having a low risk of bias related to the outcome measurement [25,28,29,31–43].

The results of the evaluation of the RCT with cross-over design is presented in S1 Fig, Supplemental Material. The study was seen as applicable to an overall high risk of bias [30]. Some concerns were evaluated referring to the randomization process and a low risk of bias was judged for the outcome measurement as well as missing outcome data.

The two non-RCTs were rated with an overall moderate risk of bias [44,45]. Bias due to missing data was judged in one study with a low [45] and in the other study with a moderate risk [44]. In addition, both studies were evaluated with a moderate risk of bias concerning the measurement of the outcome [44,45].

### Meta-analysis

16 different studies were included in meta-analysis and evaluated regarding time-domain and/or frequency-domain parameters [25,28,29,31–38,40,41,43–45]. No study was excluded because of the risk of bias assessment. Analyses of SDNN, RMSSD, LF, HF and LF/HF ratio were separated into short-term assessment (recording less than 30 minutes) and into 24-hour assessment. The results are separately presented for short-term and 24-hour assessment as well as for time-domain and frequency-domain parameters. ULF and VLF parameters were excluded from meta-analysis, as there was only one study with short-term assessment that presented available data [45]. Two other studies reported the results in a logarithmic scale for 24-hour measurements. In one of these studies, the transformed raw data showed an extreme high SD [28,36]. The parameter nLF was also excluded from meta-analysis because the significance and informative value of nHF and nLF are identical [46,47]. In all forest plots the results within the right area from the axis favour the intervention group and all results within the left area from the axis favour the control group.

### Time-domain parameter

#### Short-term

**Fig 3:**
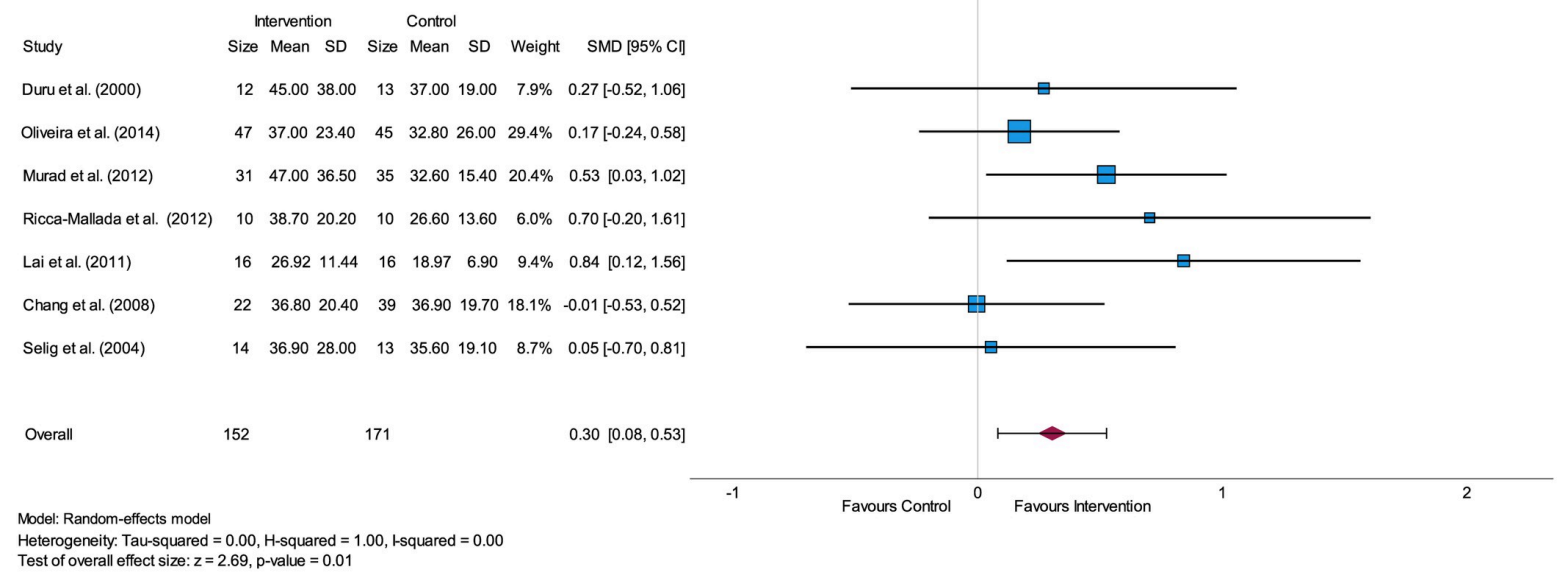
Forest plot of the time-domain parameter SDNN for short-term assessment presenting the effect of physical activity intervention vs. usual care or no intervention. SD = standard deviation, SMD = standard mean difference, CI = confidence interval, SDNN = standard deviation of Normal-to-Normal intervals.

In nearly all studies physical activity is associated with a higher mean RR-Interval, RMSSD and/or SDNN after the intervention period compared to the control group [29,31–35,41]. The analysis of RR-Interval included 121 participants in an intervention and 122 participants in a control group from five different studies [25,32,34,41,45]. Analysis of RMSSD contained 144 participants in an intervention group in comparison to the control group with 162 participants from overall six studies [29,32,33,35,41,45] and analysis of SDNN included seven studies with 152 participants in the intervention group and 171 participants in the control group [31–34,41,44,45]. Heterogeneity was moderate (I^2^ = 0.48) for RR-Interval, considerable (I^2^ = 0.69) for RMSSD and not present (I^2^ = 0.00) for SDNN. Each overall effect size was small (SMD up to 0.39) as shown in S2 and S3 Figs, Supplemental Material for RR-Interval as well as RMSSD and Fig 3 for SDNN.

#### 24-hour

**Fig 4:**
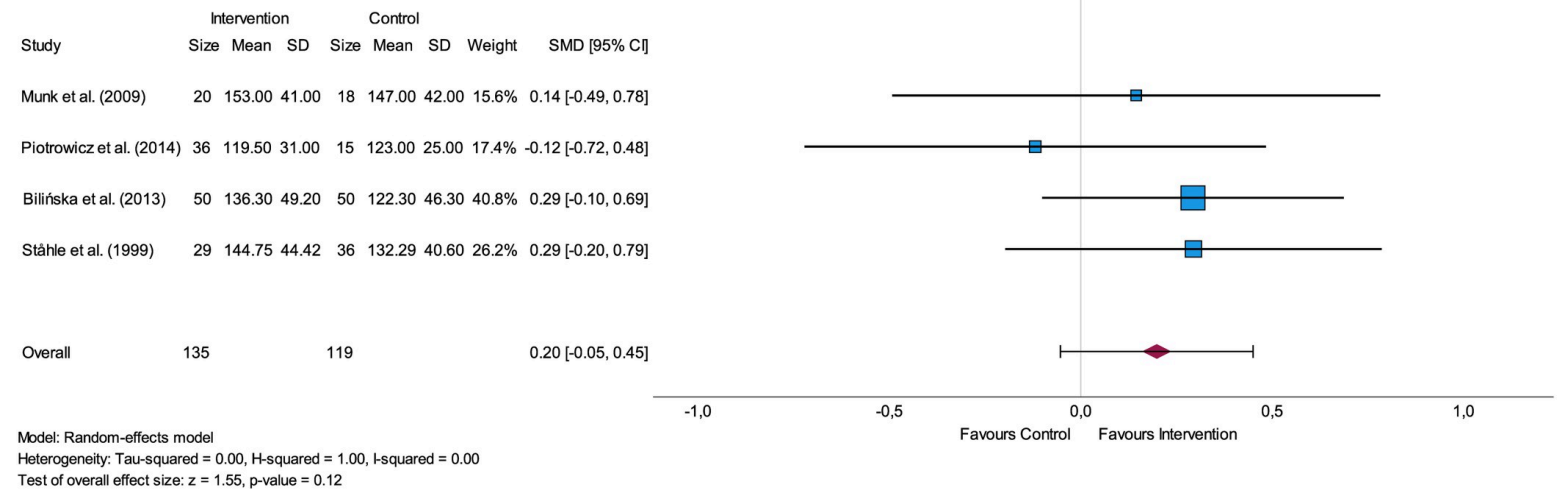
Forest plot of time-domain parameter SDNN for 24-hour assessment presenting the impact of physical activity intervention in comparison to usual care or no intervention. SD = standard deviation, SMD = standard mean difference, CI = confidence interval, SDNN = standard deviation of Normal-to-Normal intervals.

Analysis of RMSSD included 49 participants in an intervention group and 54 participants in a control group from two studies [28,36]. The analysis of SDNN included four studies and was conducted with 135 participants in the intervention and 119 participants in the control group [28,36–38]. Nearly all studies included in the meta-analysis of RMSSD and SDNN showed higher mean post-interventional values in the group assigned to physical activity [28,36,37]. Heterogeneity was not present for both parameters (I^2^ = 0.00) and the overall effect sizes were small (SMD up to 0.20). S4 Fig, Supplemental Material shows the forest plot for the parameter RMSSD and Fig 4 consecutively for SDNN.

### Frequency-domain parameter

#### Short-term

The analysis of TP included three studies with 49 participants in the intervention group in comparison to 66 participants in the control group. All three studies presented higher mean post-intervention values in the group assigned to physical activity [29,43,45]. Regarding the analysis of LF, four studies were included with a total of 64 participants as part of an intervention and 76 participants as part of a control group [29,34,43,45]. Heterogeneity showed to be moderate (I^2^ = 0.30) for TP and low (I^2^ = 0.26) for LF. The overall effect sizes were small (SMD up to 0.43), as shown in S5 and S6 Figs, Supplemental Material. The analysis of HF included six studies with 93 participants in the intervention and 109 participants in the control group [25,29,34,35,43,45]. For the parameter nHF, the analysis included 76 participants in the intervention and 91 participants in the control group from five studies [31,40,41,44,45]. Heterogeneity was considerable (I^2^ = 0.83) for HF and low (I^2^ = 0.19) for nHF. The overall effect sizes showed to be small for both, the parameter HF (SMD = 0.45) favouring the intervention group (S7 Fig, Supplemental Material) and for nHF (SMD = -0.04) favouring the control group (S8 Fig, Supplemental Material). The analysis related to LF/HF included seven studies with 155 participants joining the intervention and 152 participants joining the control group [25,29,32,34,43–45]. Heterogeneity was low (I^2^ = 0.13) and the overall effect size small (SMD = 0.19) as shown in S9 Fig, Supplemental Material.

#### 24-hour

The meta-analysis of LF, HF as well as LF/HF included four studies with 133 participants in the intervention group and 116 participants in the control group [28,36–38]. Heterogeneity was moderate for LF (I^2^ = 0.63), not present for HF (I^2^ = 0.00) and considerable for LF/HF (I^2^ = 0.73). The overall effect sizes were small (SMD up to 0.18) for LF and HF as demonstrated in S10 and S11 Figs, Supplemental Material and medium (SMD = 0.51) for LF/HF ratio (S12 Fig, Supplemental Material).

#### Congestive heart failure

**Fig 5:**
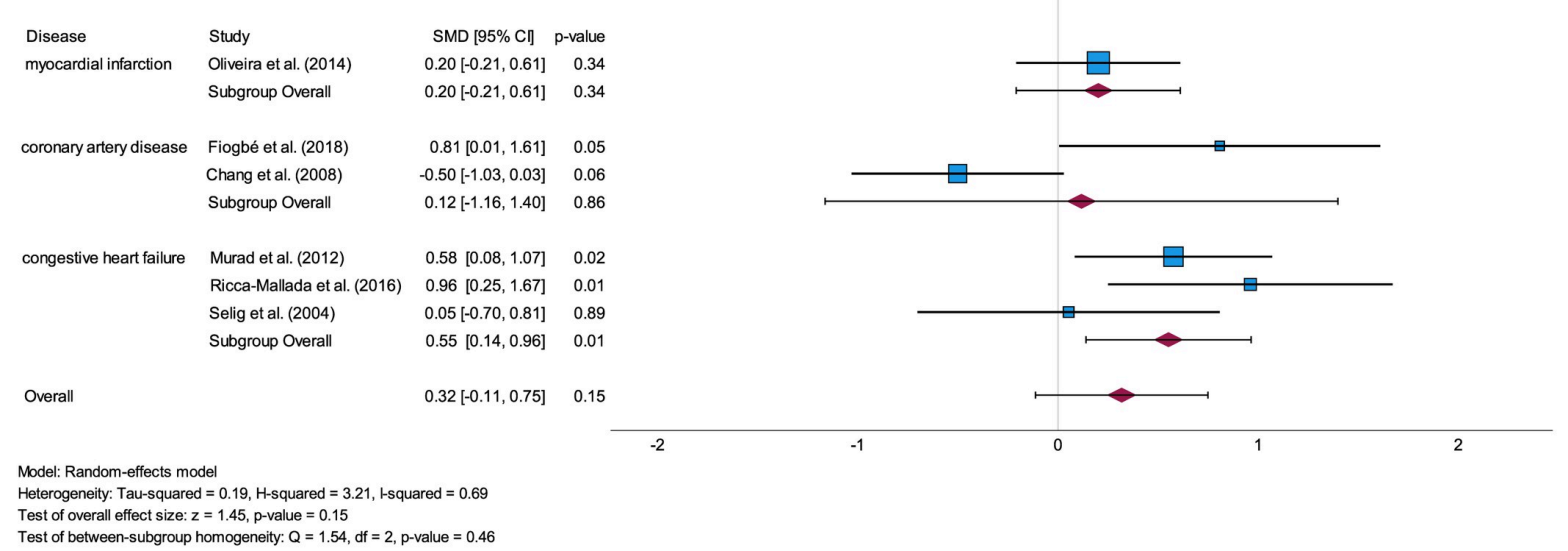
Forest plot of subgroup analysis illustrating the impact of physical activity exercises on the time-domain parameter RMSSD related to investigated heart diseases. RMSSD = root-mean-square difference of successive normal R-R intervals, SMD = standard mean difference, CI = confidence interval.

The subgroup analysis of the disease CHF included two studies for the analysis of RR-Interval [34,41], three studies for both RMSSD [33,35,41] and SDNN [33,34,41], one study for LF [34] and two studies for HF [34,35]. Of these, analysis of RMSSD (p = 0.01), SDNN (p = 0.02) and HF (p < 0.01) showed significant results for CHF. The effect sizes ranged from small (SMD = 0.44) to high (SMD = 0.98) values as summarized in S3 Table, Supplemental Material. In this regard, the results of the subgroup-homogeneity test showed a significant difference for the parameter HF (p = 0.01) between the estimated SMD for CHF and that the other subgroups. However, for the remaining investigated parameters, the results of the subgroup-homogeneity test did not show statistical significance. The results concerning the test of subgroup-homogeneity are presented in S4 Table, Supplemental Material. Additionally, studies with aerobic exercise training [33,35] or a combination of aerobic exercise training and resistance training [34] showed higher effect sizes in comparison to resistance training only [41]. Within the subgroup analysis regarding the different intervention types, aerobic exercise training presented significant results for RMSSD (p = 0.01) with a medium effect size (SMD = 0.51) and for HF (p = 0.01) with a high effect size (SMD = 1.0). The combination of aerobic and resistance training was significant for HF (p = 0.04) with a high effect size (SMD = 0.95) as can be seen in S5 Table, Supplemental Material. The forest plot, shown in Fig 5, illustrates the results of the subgroup analysis for RMSSD.

Within this subgroup analysis, studies were considered that investigated participants with CHF but without a recent or any history of myocardial infarction (MI). In addition, studies investigating CHF patients but without mentioning information regarding a past occurrence of MI were included as well. Thus, the study in that patients after a recent MI and with a new-onset heart failure were investigated, was not included in this analysis [31].

#### Coronary artery disease

Subgroup analysis related to CAD included one study for the parameter RR-Interval [45], two studies for RMSSD [29,45] as well as SDNN [44,45], TP, LF and HF [29,45]. All parameters showed no significance for the subgroup analysis in relation to the investigated disease. T’ai Chi as an intervention [45] showed lower effect sizes compared to water aerobic exercise training [29] or walking [44]. Within the subgroup analysis regarding the different intervention types, water aerobic exercise training was significant for the parameter RMSSD (p = 0.05), as well as TP (p = 0.01) and HF (p < 0.01). Especially for HF we could prove the obtained effect size as very high (SMD = 1.94) related to water aerobic exercise training. Furthermore, the results of the subgroup-homogeneity test, presented in S6 Table, Supplemental Material, showed significant differences between the estimated SMD for water aerobic exercise training compared to the SMD of the other intervention types. Specifically, significant differences were observed for the parameters HF (p < 0.001) and RMSSD (p = 0.01). Walking as an intervention type also showed significance within the subgroup analysis for one time-domain parameter, SDNN (p = 0.02).

All analyses related to CAD included at least one non-RCT [45]. Analysis of SDNN only included non-RCTs [44,45]. The conducted subgroup analysis with regard to the study design only presented significant results for RMSSD related to RCTs (p < 0.01) and SDNN also related to RCTs (p = 0.02), as shown in S7 Table, Supplemental Material. Moreover, the subgroup-homogeneity test revealed significant differences for the parameter RMSSD (p = 0.002) and HF (p = 0.05) in relation to the estimated SMD between RCTs and non-RCTs. Detailed results are shown in S8 Table, Supplemental Material. The effect sizes of the investigated non-RCTs were both small for SDNN (SMD = 0.38) favouring the intervention group and for RMSSD (SMD = -0.50) favouring the control group.

Regarding this subgroup analysis studies were considered in that all participants were diagnosed with CAD. In addition, some of these participants suffered from CHF or a history of MI.

#### Myocardial infarction

The subgroup analysis regarding MI as one type of ACS included one study for the parameter RR-Interval, as well as RMSSD [32] and two studies for the analysis of SDNN [31,32]. None of the investigated parameters were significant within their subgroup analysis. The estimated effect sizes were small for all parameters (SMD from 0.19 up to 0.31), as shown in S3 Table, Supplemental Material. In one study, the intervention group performed walking and cycling exercises [31]. This type of intervention also showed no significant result within the subgroup analysis related to the different interventions (S5 Table, Supplemental Material).

Within this subgroup analysis, studies were considered that investigated patients after a recent MI. A recent MI was defined as a period less than 2 months between the occurrence of MI and randomization process.

#### Peripheral arterial disease

The subgroup analysis linked to PAD included one study for RR-Interval, TP as well as LF [43] and two studies for analysis of HF [25,43]. As shown in S3 Table, Supplemental Material, all results regarding PAD were non-significant. Concerning the parameters RR-Interval, LF and HF the estimated effect sizes favoured the control group (SMD up to -0.26). In both studies walking exercise was performed within the intervention group [25,43]. As presented in S5 Table, Supplemental Material, this intervention showed non-significant results in the subgroup analysis regarding intervention type for the investigated parameters RR-Interval, TP, LF, and HF in relation to patients with PAD.

Regarding this subgroup analysis studies were considered, that investigated patients with presence of PAD and symptoms of intermittent claudication.

### Sensitivity analysis

The sensitivity analysis with the “mean difference” as an effect size, brought the following significant values for the short-term assessment: SDNN measured in ms (p < 0.01), TP measured in ms^2^ (p = 0.03) and HF measured in ms^2^/Hz (p < 0.01). Analysis of SDNN in ms included a total of seven studies [31–34,41,44,45], analysis of TP in ms^2^ included two studies [29,45] and analysis of HF in ms^2^/Hz contained three studies [34,35,43]. For the 24-hour assessment there were no significant results obtained. Effect sizes, standard errors and p-values are summarized in S9 Table, Supplemental Material.

## Discussion

This systematic review and meta-analysis evaluated the impact of different physical training interventions on HRV compared to no intervention or usual care including RCTs and non-RCTs. Multiple CVDs were combined in a single meta-analysis investigating different parameters of HRV. The findings of this analysis showed changes in several time-domain and frequency-domain parameters of HRV. In particular, beneficial effects were found related to RR-Interval, SDNN and including the subgroup analysis also to RMSSD, TP and HF for short-term ECG- and HR- assessment, as there were significant differences between the intervention and control group. Concerning the 24-hour assessment, benefits were solely found for the parameter LF/HF. It was expected that the parameter RMSSD and HF from 24-hour assessment would show similar results in the meta-analysis as they correlate with each other. Both forest plots illustrated no significant changes between the intervention and control group. Nevertheless, the analysis of RMSSD only included two studies and the analysis of HF included four studies.

The results showed that patients with CHF benefit most from regular physical activity sessions, compared to patients with PAD, CAD or patients after MI. It has been pointed out that significant results were obtained in at least one time-domain as well as frequency-domain parameter for CHF. Aerobic exercise training as well as water aerobic exercise training both achieved better results for various investigated parameters, in particular the frequency-domain parameters, compared to the other forms of physical activity. This is also shown in the results related to RMSSD and CHF. The researched studies, in which participants underwent aerobic exercise training, demonstrated larger effect sizes compared to the study involving resistance training as intervention [33,35,41]. In addition, the higher impact of aerobic exercise training in comparison to resistance training is also present for RR-Interval and SDNN. The related effect sizes are always higher in comparison to the effect size concerning resistance training for CHF patients. In a previously published systematic review similar results are shown for CHF patients: Aerobic exercise training, performed under certain conditions, can improve the autonomic function [48]. Another observation regarding the impact of aerobic exercise training and CHF patients is related to the intervention period. The estimated effect size for RMSSD is higher in the analyzed study that examined the impact of aerobic exercise training in CHF patients over a 24-week intervention period compared to the study with a 16-week intervention period [33,35] This implicates that the effect of aerobic training on the time-domain parameter of HRV can increase with time. The performed aerobic exercise training in the included studies consisted of a warm-up period followed by different exercises (i.e. walking on a track or on a treadmill, cycling) and a cool-down phase [32,33,35]. The results of another previously published systematic review also showed significant changes in HRV through conventional drugs, biobehavioral therapy and exercise training for patients with CAD [49]. Thus, exercise training can be seen as an alternative to the conventional therapy strategies. Regular exercise training improves the cardiovascular system in general and is an important component of primary and secondary prevention concerning coronary heart disease [20]. These benefits for patients with CAD are observed especially with regard to water aerobic exercise training: Although the investigated study had a much shorter intervention period of 16 weeks, the obtained effect sizes linked to water aerobic exercise training as an intervention were higher in all cases in comparison to the effect sizes related to the study with T’ai Chi as physical training [29,45]. Two other previously published reviews showed that exercise training in general improves several parameters of HRV in patients after MI [50,51]. However, our results obtained from the subgroup analysis related to MI and PAD as well were not significant even though differences between the intervention and control group were observed.

Subgroup analysis concerning the study design showed no significant results in non-RCTs. However, the results for RCTs were significant for RMSSD and SDNN. The probability for unequal distributions is lower in RCTs compared to non-RCTs as the participants are not able to choose between the intervention and the control group. Thus, those results can be seen as less affected compared to results from non-RCTs.

The sensitivity analysis with mean difference as an effect size showed significant results for SDNN measured in ms, TP in ms^2^ and HF in ms^2^/Hz for short-term ECG- or HR-recording but no significant results related to 24-hour ECG recording. However, all results indicate a change between the intervention and control group. Thus, physical activity leads to changes in all investigated parameters.

The conducted meta-analysis is limited. In some studies the baseline values of the control group were higher for several parameters compared to the intervention group, as observed for the parameters SDNN and RMSSD in at least one included study [32]. This could have affected the results related to the estimated effect sizes. Also, the sample sizes of the included studies were rather small and the intervention periods were not fully comparable due to the different disclosures. The included studies for 24-hour assessment were also not comparable to each other concerning the investigated diseases and the training methods. This caused a missing subgroup analysis for this assessment method. Furthermore, the distribution of studies within the conducted subgroup analyses was not uniform, with varying numbers across groups. Additionally, all our meta-analyses encompassed fewer than 10 studies each. Consequently, we were unable to assess publication bias using a funnel plot, and we did not conduct a search for grey literature to address potential concerns related to publication bias. This emphasizes the importance of interpreting the results with caution. Regarding studies identified from clinical trial registries without accessible or published results, it could be possible that the results were presented in a study report without reference to the study identification number used to identify the results via Google. Finally, the meta-analysis included RCTs as well as non-RCTs, however only the RCTs are known as the gold standard [52].

Despite the mentioned limitations, this meta-analysis showed that physical activity has a positive impact on HRV especially in patients with CHF. However, clinical decision cannot be solely guided on the presented results. Benefits of regular physical exercise training need to be investigated in further RCTs with higher amounts of participants for each analyzed heart disease, as we are in need of more studies within the analyzed subgroups. While this conducted study implies the potential effectiveness of aerobic exercise training, further investigation is needed to determine the optimal training type and intensity for the analyzed CVD. Additional valuable studies are essential to explore the long-term effects of physical activity on HRV, comprehensively describe and understand the underlying mechanisms, and systematically identify and assess confounding factors (e.g., medication use, nutrition, body weight, etc.) that might influence the outcomes, whether negatively or positively. In future studies, it is advisable to consider other CVD such as cerebrovascular diseases, rheumatic heart diseases, or congenital heart diseases. There is a possibility that various physical activities could offer greater benefits for different CVD, and exploring these possibilities would be valuable.

## Supporting information

S1 File (DOCX)

S1 Fig (TIF)

S2 Fig (TIFF)

S3 Fig (TIFF)

S4 Fig (TIFF)

S5 Fig (TIFF)

S6 Fig (TIFF)

S7 Fig (TIFF)

S8 Fig (TIFF)

S9 Fig (TIFF)

S10 Fig (TIFF)

S11 Fig (TIFF)

S12 Fig (TIFF)

## Abbreviations

ACS: acute coronary syndrome
CAD: coronary artery disease
CHF: congestive heart failure
CVD: cardiovascular diseases
ECG: electrocardiogram
HF: high-frequency power
HR: heart rate
HRV: heart rate variability
IHD: ischemic heart disease
LF: low-frequency power
MeSH: Medical Subject Headings
MI: myocardial infarction
nHF: HF in normalized units
nLF: LF in normalized units
PAD: peripheral arterial disease
PRISMA: Preferred Reporting of Items for Systematic Reviews and Meta-analyses
PROSPERO: International Prospective Register of Systematic Reviews
RCT: randomized controlled trial
RMSSD: root-mean-square difference of successive normal R-R intervals
SD: standard deviation
SDNN: standard deviation of Normal-to-Normal intervals
SMD: standard mean difference
TP: total power
ULF: ultra-low frequency bands
VLF: very-low frequency bands

## References

[1] Roth GA, Mensah GA, Johnson CO, Addolorato G, Ammirati E, Baddour LM, et al. Global Burden of Cardiovascular Diseases and Risk Factors, 1990–2019: Update From the GBD 2019 Study. J Am Coll Cardiol. 2020;76(25):2982–3021. doi: 10.1016/j.jacc.2020.11.010 ; PubMed Central PMCID: PMC7755038. 33309175 PMC7755038

[2] Anderson L, Thompson DR, Oldridge N, Zwisler AD, Rees K, Martin N, et al. Exercise-based cardiac rehabilitation for coronary heart disease. Cochrane Database Syst Rev. 2016;2016(1):CD001800. Epub 20160105. doi: 10.1002/14651858.CD001800.pub3 ; PubMed Central PMCID: PMC6491180. 26730878 PMC6491180

[3] Dibben G, Faulkner J, Oldridge N, Rees K, Thompson DR, Zwisler AD, et al. Exercise-based cardiac rehabilitation for coronary heart disease. Cochrane Database Syst Rev. 2021;11(11):CD001800. Epub 20211106. doi: 10.1002/14651858.CD001800.pub4 ; PubMed Central PMCID: PMC8571912. 34741536 PMC8571912

[4] Long L, Mordi IR, Bridges C, Sagar VA, Davies EJ, Coats AJ, et al. Exercise-based cardiac rehabilitation for adults with heart failure. Cochrane Database Syst Rev. 2019;1(1):CD003331. Epub 20190129. doi: 10.1002/14651858.CD003331.pub5 ; PubMed Central PMCID: PMC6492482. 30695817 PMC6492482

[5] Kamiya K, Sato Y, Takahashi T, Tsuchihashi-Makaya M, Kotooka N, Ikegame T, et al. Multidisciplinary Cardiac Rehabilitation and Long-Term Prognosis in Patients With Heart Failure. Circ Heart Fail. 2020;13(10):e006798. Epub 20200928. doi: 10.1161/CIRCHEARTFAILURE.119.006798 . 32986957

[6] Cole CR, Blackstone EH, Pashkow FJ, Snader CE, Lauer MS. Heart-rate recovery immediately after exercise as a predictor of mortality. N Engl J Med. 1999;341(18):1351–7. doi: 10.1056/NEJM199910283411804 . 10536127

[7] Mongin D, Chabert C, Extremera MG, Hue O, Courvoisier DS, Carpena P, et al. Decrease of heart rate variability during exercise: An index of cardiorespiratory fitness. PLoS One. 2022;17(9):e0273981. Epub 20220902. doi: 10.1371/journal.pone.0273981 ; PubMed Central PMCID: PMC9439241. 36054204 PMC9439241

[8] Manresa-Rocamora A, Sarabia JM, Guillen-Garcia S, Perez-Berbel P, Miralles-Vicedo B, Roche E, et al. Heart Rate Variability-Guided Training for Improving Mortality Predictors in Patients with Coronary Artery Disease. Int J Environ Res Public Health. 2022;19(17). Epub 20220823. doi: 10.3390/ijerph191710463 ; PubMed Central PMCID: PMC9518028. 36078179 PMC9518028

[9] Tiwari R, Kumar R, Malik S, Raj T, Kumar P. Analysis of Heart Rate Variability and Implication of Different Factors on Heart Rate Variability. Curr Cardiol Rev. 2021;17(5):e160721189770. doi: 10.2174/1573403X16999201231203854 ; PubMed Central PMCID: PMC8950456. 33390146 PMC8950456

[10] Jarczok MN, Weimer K, Braun C, Williams DP, Thayer JF, Gundel HO, et al. Heart rate variability in the prediction of mortality: A systematic review and meta-analysis of healthy and patient populations. Neurosci Biobehav Rev. 2022;143:104907. Epub 20221013. doi: 10.1016/j.neubiorev.2022.104907 . 36243195

[11] Shibasaki K, Ogawa S, Yamada S, Ouchi Y, Akishita M. Role of autonomic nervous activity, as measured by heart rate variability, on the effect of mortality in disabled older adults with low blood pressure in long-term care. Geriatr Gerontol Int. 2018;18(8):1153–8. Epub 20180411. doi: 10.1111/ggi.13328 . 29644805

[12] van Boven AJ, Jukema JW, Haaksma J, Zwinderman AH, Crijns HJ, Lie KI. Depressed heart rate variability is associated with events in patients with stable coronary artery disease and preserved left ventricular function. REGRESS Study Group. Am Heart J. 1998;135(4):571–6. doi: 10.1016/s0002-8703(98)70269-8 . 9539469

[13] Huikuri HV, Stein PK. Heart rate variability in risk stratification of cardiac patients. Prog Cardiovasc Dis. 2013;56(2):153–9. Epub 20130812. doi: 10.1016/j.pcad.2013.07.003 . 24215747

[14] Shaffer F, Ginsberg JP. An Overview of Heart Rate Variability Metrics and Norms. Front Public Health. 2017;5:258. Epub 20170928. doi: 10.3389/fpubh.2017.00258 ; PubMed Central PMCID: PMC5624990. 29034226 PMC5624990

[15] Umetani K, Singer DH, McCraty R, Atkinson M. Twenty-four hour time domain heart rate variability and heart rate: relations to age and gender over nine decades. J Am Coll Cardiol. 1998;31(3):593–601. doi: 10.1016/s0735-1097(97)00554-8 . 9502641

[16] Zhao R, Li D, Zuo P, Bai R, Zhou Q, Fan J, et al. Influences of age, gender, and circadian rhythm on deceleration capacity in subjects without evident heart diseases. Ann Noninvasive Electrocardiol. 2015;20(2):158–66. Epub 20140812. doi: 10.1111/anec.12189 ; PubMed Central PMCID: PMC4407920. 25112779 PMC4407920

[17] Reyes del Paso GA, Langewitz W, Mulder LJ, van Roon A, Duschek S. The utility of low frequency heart rate variability as an index of sympathetic cardiac tone: a review with emphasis on a reanalysis of previous studies. Psychophysiology. 2013;50(5):477–87. Epub 20130227. doi: 10.1111/psyp.12027 . 23445494

[18] Shaffer F, McCraty R, Zerr CL. A healthy heart is not a metronome: an integrative review of the heart’s anatomy and heart rate variability. Front Psychol. 2014;5:1040. Epub 20140930. doi: 10.3389/fpsyg.2014.01040 ; PubMed Central PMCID: PMC4179748. 25324790 PMC4179748

[19] Villella M, Villella A. Exercise and cardiovascular diseases. Kidney Blood Press Res. 2014;39(2–3):147–53. Epub 20140729. doi: 10.1159/000355790 . 25117881

[20] Lavie CJ, Thomas RJ, Squires RW, Allison TG, Milani RV. Exercise training and cardiac rehabilitation in primary and secondary prevention of coronary heart disease. Mayo Clin Proc. 2009;84(4):373–83. doi: 10.1016/S0025-6196(11)60548-X ; PubMed Central PMCID: PMC2665984. 19339657 PMC2665984

[21] Page MJ, McKenzie JE, Bossuyt PM, Boutron I, Hoffmann TC, Mulrow CD, et al. The PRISMA 2020 statement: an updated guideline for reporting systematic reviews. BMJ. 2021;372:n71. Epub 20210329. doi: 10.1136/bmj.n71 ; PubMed Central PMCID: PMC8005924. 33782057 PMC8005924

[22] Clark JM, Sanders S, Carter M, Honeyman D, Cleo G, Auld Y, et al. Improving the translation of search strategies using the Polyglot Search Translator: a randomized controlled trial. J Med Libr Assoc. 2020;108(2):195–207. Epub 20200401. doi: 10.5195/jmla.2020.834 ; PubMed Central PMCID: PMC7069833. 32256231 PMC7069833

[23] Sterne JAC, Savovic J, Page MJ, Elbers RG, Blencowe NS, Boutron I, et al. RoB 2: a revised tool for assessing risk of bias in randomised trials. BMJ. 2019;366:l4898. Epub 20190828. doi: 10.1136/bmj.l4898 . 31462531

[24] Sterne JA, Hernan MA, Reeves BC, Savovic J, Berkman ND, Viswanathan M, et al. ROBINS-I: a tool for assessing risk of bias in non-randomised studies of interventions. BMJ. 2016;355:i4919. Epub 20161012. doi: 10.1136/bmj.i4919 ; PubMed Central PMCID: PMC5062054. 27733354 PMC5062054

[25] Sandercock GR, Hodges LD, Das SK, Brodie DA. The impact of short term supervised and home-based walking programmes on heart rate variability in patients with peripheral arterial disease. J Sports Sci Med. 2007;6(4):471–6. Epub 20071201. ; PubMed Central PMCID: PMC3794487. 24149480 PMC3794487

[26] Higgins JPT LT, Deeks JJ (editors). Chapter 6: Choosing effect measures and computing estimates of effect. In: Higgins JPT TJ, Chandler J, Cumpston M, Li T, Page MJ, Welch VA, editor. Cochrane Handbook for Systematic Reviews of Interventions version 6.3: Cochrane; 2022.

[27] Higgins JP, White IR, Anzures-Cabrera J. Meta-analysis of skewed data: combining results reported on log-transformed or raw scales. Stat Med. 2008;27(29):6072–92. doi: 10.1002/sim.3427 ; PubMed Central PMCID: PMC2978323. 18800342 PMC2978323

[28] Stâhle A, Nordlander R, Bergfeldt L. Aerobic group training improves exercise capacity and heart rate variability in elderly patients with a recent coronary event. A randomized controlled study. European heart journal. 1999;20(22):1638–46. doi: 10.1053/euhj.1999.1715 10543927

[29] Fiogbé E, Ferreira R, Sindorf MAG, Tavares SA, de Souza KP, de Castro Cesar M, et al. Water exercise in coronary artery disease patients, effects on heart rate variability, and body composition: A randomized controlled trial. Physiotherapy research international: the journal for researchers and clinicians in physical therapy. 2018;23(3):e1713. doi: 10.1002/pri.1713 29542251

[30] Adamopoulos S, Piepoli M, McCance A, Bernardi L, Rocadaelli A, Ormerod O, et al. Comparison of different methods for assessing sympathovagal balance in chronic congestive heart failure secondary to coronary artery disease. The American journal of cardiology. 1992;70(20):1576–82. doi: 10.1016/0002-9149(92)90460-g 1466326

[31] Duru F, Candinas R, Dziekan G, Goebbels U, Myers J, Dubach P. Effect of exercise training on heart rate variability in patients with new-onset left ventricular dysfunction after myocardial infarction. American heart journal. 2000;140(1):157–61. doi: 10.1067/mhj.2000.106606 10874279

[32] Oliveira NL, Ribeiro F, Teixeira M, Campos L, Alves AJ, Silva G, et al. Effect of 8-week exercise-based cardiac rehabilitation on cardiac autonomic function: A randomized controlled trial in myocardial infarction patients. Am Heart J. 2014;167(5):753–61 e3. Epub 20140217. doi: 10.1016/j.ahj.2014.02.001 . 24766987

[33] Murad K, Brubaker PH, Fitzgerald DM, Morgan TM, Goff DCJ, Soliman EZ, et al. Exercise training improves heart rate variability in older patients with heart failure: a randomized, controlled, single-blinded trial. Congestive heart failure (Greenwich, Conn). 2012;18(4):192–7. doi: 10.1111/j.1751-7133.2011.00282.x 22536936 PMC3400715

[34] Ricca-Mallada R, Migliaro ER, Piskorski J, Guzik P. Exercise training slows down heart rate and improves deceleration and acceleration capacity in patients with heart failure. Journal of electrocardiology. 2012;45(3):214–9. doi: 10.1016/j.jelectrocard.2012.01.002 22341740

[35] Ricca-Mallada R, Migliaro ER, Silvera G, Chiappella L, Frattini R, Ferrando-Castagnetto F. Functional outcome in chronic heart failure after exercise training: Possible predictive value of heart rate variability. Annals of physical and rehabilitation medicine. 2016;60(2):87–94. doi: 10.1016/j.rehab.2016.12.003 28131566

[36] Munk PS, Butt N, Larsen AI. High-intensity interval exercise training improves heart rate variability in patients following percutaneous coronary intervention for angina pectoris. International journal of cardiology. 2010;145(2):312–4. doi: 10.1016/j.ijcard.2009.11.015 19962772

[37] Bilińska M, Kosydar-Piechna M, Mikulski T, Piotrowicz E, Gasiorowska A, Piotrowski W, et al. Influence of aerobic training on neurohormonal and hemodynamic responses to head-up tilt test and on autonomic nervous activity at rest and after exercise in patients after bypass surgery. Cardiology Journal. 2013;20(1):17–24. doi: 10.5603/CJ.2013.0004 23558806

[38] Piotrowicz E, Piotrowski W, Piotrowicz R. Influence of home-based telemonitored Nordic walking training on autonomic nervous system balance in heart failure patients Arch Med Sci. 2014;11(6):1205–12. Epub doi: 10.5114/aoms.2015.56346 PubMed Central PMCID: PMC4697054. PMC4697054 26788081

[39] Leicht AS, Crowther RG, Golledge J. Influence of peripheral arterial disease and supervised walking on heart rate variability. Journal of vascular surgery. 2011;54(5):1352–9. doi: 10.1016/j.jvs.2011.05.027 21784603

[40] Tamburus NY, Paula RFL, Kunz VC, César MC, Moreno MA, da Silva E. Interval training based on ventilatory anaerobic threshold increases cardiac vagal modulation and decreases high-sensitivity c-reative protein: randomized clinical trial in coronary artery disease. Brazilian journal of physical therapy. 2015;19(6):441–50. doi: 10.1590/bjpt-rbf.2014.0124 26647745 PMC4668337

[41] Selig SE, Carey MF, Menzies DG, Patterson J, Geerling RH, Williams AD, et al. Moderate-intensity resistance exercise training in patients with chronic heart failure improves strength, endurance, heart rate variability, and forearm blood flow. Journal of cardiac failure. 2004;10(1):21–30. doi: 10.1016/s1071-9164(03)00583-9 14966771

[42] Kiilavuori K, Toivonen L, Näveri H, Leinonen H. Reversal of autonomic derangements by physical training in chronic heart failure assessed by heart rate variability. European heart journal. 1995;16(4):490–5. doi: 10.1093/oxfordjournals.eurheartj.a060941 7671894

[43] Brenner IKM, Brown CA, Hains SJM, Tranmer J, Zelt DT, Brown PM. Low-Intensity Exercise Training Increases Heart Rate Variability in Patients With Peripheral Artery Disease. Biological research for nursing. 2020;22(1):24–33. doi: 10.1177/1099800419884642 31684758

[44] Lai F-C, Tu S-T, Huang C-H, Jeng C. A home-based exercise program improves heart rate variability and functional capacity among postmenopausal women with coronary artery disease. The Journal of cardiovascular nursing. 2011;26(2):137–44. doi: 10.1097/JCN.0b013e3181ed9424 21076311

[45] Chang R-Y, Koo M, Yu Z-R, Kan C-B, Chu I-T, Hsu C-T, et al. The effect of t’ai chi exercise on autonomic nervous function of patients with coronary artery disease. Journal of alternative and complementary medicine (New York, NY). 2008;14(9):1107–13. doi: 10.1089/acm.2008.0166 18991518

[46] Heathers JA. Everything Hertz: methodological issues in short-term frequency-domain HRV. Front Physiol. 2014;5:177. Epub 20140507. doi: 10.3389/fphys.2014.00177 ; PubMed Central PMCID: PMC4019878. 24847279 PMC4019878

[47] Burr RL. Interpretation of normalized spectral heart rate variability indices in sleep research: a critical review. Sleep. 2007;30(7):913–9. doi: 10.1093/sleep/30.7.913 ; PubMed Central PMCID: PMC1978375. 17682663 PMC1978375

[48] Hsu CY, Hsieh PL, Hsiao SF, Chien MY. Effects of Exercise Training on Autonomic Function in Chronic Heart Failure: Systematic Review. Biomed Res Int. 2015;2015:591708. Epub 20151012. doi: 10.1155/2015/591708 ; PubMed Central PMCID: PMC4620239. 26543861 PMC4620239

[49] Nolan RP, Jong P, Barry-Bianchi SM, Tanaka TH, Floras JS. Effects of drug, biobehavioral and exercise therapies on heart rate variability in coronary artery disease: a systematic review. Eur J Cardiovasc Prev Rehabil. 2008;15(4):386–96. doi: 10.1097/HJR.0b013e3283030a97 . 18677161

[50] Oliveira NL, Ribeiro F, Alves AJ, Teixeira M, Miranda F, Oliveira J. Heart rate variability in myocardial infarction patients: effects of exercise training. Rev Port Cardiol. 2013;32(9):687–700. Epub 20130830. doi: 10.1016/j.repc.2013.02.010 . 23993292

[51] Routledge FS, Campbell TS, McFetridge-Durdle JA, Bacon SL. Improvements in heart rate variability with exercise therapy. Can J Cardiol. 2010;26(6):303–12. doi: 10.1016/s0828-282x(10)70395-0 ; PubMed Central PMCID: PMC2903986. 20548976 PMC2903986

[52] Cuschieri S. The CONSORT statement. Saudi J Anaesth. 2019;13(Suppl 1):S27–S30. doi: 10.4103/sja.SJA_559_18 ; PubMed Central PMCID: PMC6398298. 30930716 PMC6398298

