# Supplementary material for: Beneficial impacts of physical activity on heart rate variability: A systematic review and meta-analysis
Source: PLoS One. 2024 Apr 5;19(4):e0299793. doi: 10.1371/journal.pone.0299793 (PMC10997132; doi:10.1371/journal.pone.0299793)
Supplement: S1 File — (DOCX) [file pone.0299793.s001.docx]

Authors

Ouahiba El-Malahi, Darya Mohajeri, Raluca Mincu, Alexander Bäuerle, Korbinian Rothenaicher, Ramtin Knuschke, Christos Rammos, Tienush Rassaf, Julia Lortz

Supplemental material

Beneficial impacts of physical activity on heart rate variability: A systematic review and meta-analysis

**Index**

[Method 2](#_Toc156402881)

[Search strategy 2](#_Toc156402882)

[Results 5](#_Toc156402883)

[Study selection and characteristics 5](#_Toc156402884)

[Risk of bias 10](#_Toc156402885)

[Meta-analysis 10](#_Toc156402886)

[Time-domain parameter 10](#_Toc156402887)

[Frequency-domain parameter 11](#_Toc156402888)

[Subgroup analysis 14](#_Toc156402889)

[References 18](#_Toc156402890)

# **Method**

## **Search strategy**

**Table S1. Search strings and number of results**

| **Search process** | **Source and date** | **Search term** | **Number of results** |
| --- | --- | --- | --- |
| **1** | Pubmed  16/09/2022 | (training OR exercise OR program OR “physical activity” OR “cardiac rehabilitation”) AND ("coronary artery disease" OR “chronic ischemic heart disease” OR “acute coronary syndrome“ OR “heart failure“ OR “peripheral arterial disease“) AND "heart rate variability" | 319 |
| **2** |  | ("Resistance Training"[Mesh] OR "Exercise"[Mesh] OR "Physical Conditioning, Human"[Mesh]) AND ("Coronary Artery Disease"[Mesh] OR "Myocardial Ischemia"[Mesh] OR "Acute Coronary Syndrome"[Mesh] OR "Heart Failure"[Mesh] OR "Peripheral Arterial Disease"[Mesh]) AND "heart rate variability" | 84 |
| **3** | Embase  16/09/2022 | (training OR exercise OR program OR 'physical activity' OR 'cardiac rehabilitation') AND ('coronary artery disease' OR 'chronic ischemic heart disease' OR 'acute coronary syndrome' OR 'heart failure' OR 'peripheral arterial disease') AND 'heart rate variability'  *(converted via Polyglot[1])* | 147 |
| **4** |  | ('Resistance Training'/exp OR Exercise/exp OR 'Physical Conditioning, Human'/exp) AND ('Coronary Artery Disease'/exp OR 'Myocardial Ischemia'/exp OR 'Acute Coronary Syndrome'/exp OR 'Heart Failure'/exp OR 'Peripheral Arterial Disease'/exp) AND 'heart rate variability'  *(converted via Polyglot[1])* | 60 |
|  |  | ('Resistance Training'/exp OR Exercise/exp) AND ('Coronary Artery Disease'/exp OR 'Heart Muscle Ischemia'/exp OR 'Acute Coronary Syndrome'/exp OR 'Heart Failure'/exp OR 'Peripheral Occlusive Artery Disease'/exp) AND 'heart rate variability'  *(search term matched with Emtree)* |  |
| **5** | Cochrane Library  18/09/2022 | (training OR exercise OR program OR “physical activity” OR “cardiac rehabilitation”) AND (“coronary artery disease” OR “chronic ischemic heart disease” OR “acute coronary syndrome” OR “heart failure” OR “peripheral arterial disease”) AND “heart rate variability”  *(converted via Polyglot[1])* | 221 |
| **6** | ClinicalTrials.gov  19/09/2022 | *search field “Condition or disease”:*  ("coronary artery disease" OR “chronic ischemic heart disease” OR “acute coronary syndrome“ OR “heart failure“ OR “peripheral arterial disease“)  *search field “Other terms”:*  (training OR exercise OR program OR “physical activity” OR “cardiac rehabilitation”) AND “heart rate variability” | 100 |
| **7** | German Clinical Trials Register (DRKS)  19/09/2022 | (training OR exercise OR program OR 'physical activity' OR 'cardiac rehabilitation') AND ('coronary artery disease' OR 'chronic ischemic heart disease' OR 'acute coronary syndrome' OR 'heart failure' OR 'peripheral arterial disease') AND 'heart rate variability' | 17 |
| **8** | ICTRP  19/09/2022 | (training OR exercise OR program OR “physical activity” OR “cardiac rehabilitation”) AND (“coronary artery disease” OR “chronic ischemic heart disease” OR “acute coronary syndrome“ OR “heart failure“ OR “peripheral arterial disease“) AND "heart rate variability" | 11 |
| **9** | ISRCTN registry  21/09/2022 | (training OR exercise OR program OR “physical activity” OR “cardiac rehabilitation”) AND ("coronary artery disease" OR “chronic ischemic heart disease” OR “acute coronary syndrome“ OR “heart failure“ OR “peripheral arterial disease“) AND "heart rate variability" | 12 |
| **10** | AHA/ASA Journals  22/09/2022 | (training OR exercise OR program OR physical activity OR cardiac rehabilitation) AND ("coronary artery disease" OR chronic ischemic heart disease OR acute coronary syndrome OR heart failure OR peripheral arterial disease) AND "heart rate variability" AND (study OR trial) | 34 |
| **11** | The American Journal of Cardiology  22/09/2022 | (training OR exercise OR program OR physical activity OR cardiac rehabilitation) AND ("coronary artery disease" OR chronic ischemic heart disease OR acute coronary syndrome OR heart failure OR peripheral arterial disease) AND "heart rate variability" AND (study OR trial) | 14 |
| **12** | International Journal of Cardiology  22/09/2022 | (training OR exercise OR program OR physical activity OR cardiac rehabilitation) AND ("coronary artery disease" OR chronic ischemic heart disease OR acute coronary syndrome OR heart failure OR peripheral arterial disease) AND "heart rate variability" AND (study OR trial) | 32 |
| **Total** | | | **1051** |
| **Total after removing duplicates** | | | **706** |

# **Results**

## **Study selection and characteristics**

**Table S2. Characteristics of ongoing and completed studies without accessible or published results**

| **Study name** | **Methods** | **Participants** | **Interventions** | **Outcomes** | **Starting date** | **Notes** |
| --- | --- | --- | --- | --- | --- | --- |
| “Aerobic Exercise Training & the Autonomic System In Patients After Myocardial Infarction or Stroke”[2]  (NCT00259948) | Randomized, interventional clinical trial, factorial assignment | Adults 20 to 80 years after myocardial Infarction and percutaneous coronary interventions or after a first stroke (up to two months) with a NIH Stroke Score of 6-20; Healthy people without know cardiac disease for a second control group | Intervention: aerobic exercise training for 3 months (2 sessions per week with 60min/session)  Control I: no physical training  Control II: aerobic exercise training | Heart rate variability, blood pressure, blood sugar level, triglycerides, HDL, LDL, CRP and aerobic physical fitness | January 2006 | E-mail (20/10/2022) sent to get more information about the trial and its results. E-mail returned as mailboxes are not available anymore. Other persons from the Hadassah Medical Organization also contacted, though no personal response. |
| “Effects of Baduanjin Exercise on Heart Failure Patients”[3]  (NCT04981197) | Randomized, interventional clinical trial, parallel assignment | Adults ≥ 20 years with stable heart failure (New York Heart Association I and II), able to speak Chinese and to use video devices at home | Intervention: Baduanjin exercise for 12 weeks  Control: usual care | Changes in the modified Piper Fatigue Scale scores, the Minnesota Living with Heart Failure Questionnaire scores, the Pittsburgh Sleep Quality Index scores, the Hospital Anxiety and Depression Scale scores and heart rate variability | December 2013 | No contact e-mail adress found as well as results for this trial. |
| “Exercise to Improve Sleep in Heart Failure”[4]  (NCT00194701) | Randomized, interventional clinical trial, single group assignment | Adults ≥ 18 years with no dyspnea at rest but fatigue, dyspnea, or anginal pain or discomfort with either ordinary or less physical activity, with an evidence of one clinical episode of HF and stable HF | Intervention: walking exercise for 16 weeks (up to 5 times per week, up to 30min/session)  Control: no details | Measures of sleep (somnographic and self-reported), physiologic measures of cardiac function, self-reported holistic and health-related quality of life | April 1998 | No contact e-mail adress found as well as results for this trial. |
| “Home Walking Exercise Training in Advanced Heart Failure”[5]  (NCT00012883) | Randomized, interventional clinical trial, parallel assignment | Adults 18 to 80 years with stable heart failure related to the last three months | Intervention: home-walking exercise program for 12 weeks (5 times per week, up to 60min/session)  Control: no details | Assessment (pre- and post-study) of: peak VO_2_ and ventilatory threshold, 6-minute walk test, Heart Failure Functional Status Inventory, Cardiac Quality of life Index, SF-36, Dyspnea-Fatigue Index, norepinephrine and heart rate variability | December 2001 | E-Mail adress was found via Google and an e-mail was sent (22/10/2022) for more information about the trial and its results.  However no response reveived. |
| “Self Selected Exercise Intensity in PAD Patients”[6]  (NCT04333615) | Randomized, interventional clinical trial,  crossover assignment | Adults between 40 and 90 years with peripheral artery disease in at least one limb (ankle brachial index <0.9), claudication symptoms) six-minute walking test), able to walk at least 90m without interruption and to perform the exercise sessions | Self-selected exercise session: 30min exercise with self-selected intensity  Walking with pain exercise session: 30min exercise with series of 3 to 5min feeling moderate or maximum pain  Control session: 30min resting on treadmill | Cerebral perfusion, heart rate variability, feeling scale, subjective perceived effort and cognitive function | September 2021 | E-Mail adress was found via Google and contacted (22/10/2022) for more information about the trial and its results. Response received (24/10/2022): Study is finishing and data is currently analyzing. |
| “Promotion of Physical Activity in primary health care”[7]  (RBR-3y8w4w3) | Randomized, interventional clinical trial, parallel assignment | Adults 18 to 80 years using the Basic Health Units or Family Health Units of Bauru with no decompensated cardiovascular and/or pulmonary disease, no contraindications for physical exercises (i.e. acute myocardial infarction, unstable angina, symptomatic severe aortic stenosis, acute pulmonary embolism or pulmonary infarction) | Intervention I: remotely supervised physical activity (social media)  Intervention II: remotely supervised physical activity (mobile phone)  intervention III: supervised physical activity (face-to-face)  Control: no supervised physical activity | Arterial stiffness, autonomic cardiac modulation, cardiovascular health profile, endothelial function, functional capacity, health costs, morbidity, mortality, physical fitness, quality of life and mood | April 2020 (first enrollment) | Study results could give important information, however not clearly described which kind of heart disease will be investigated next to the other diseases and non-supervised home training in the control group is not excluded. E-mail (22/10/2022) sent to get more information. However, no response received. |
| “Comparison of high and moderate intensity cardiac rehabilitation after CABG”[8]  (IRCT20220326054352N1) | Randomized, interventional clinical trial, parallel assignment | Adults 35 to 60 years with sinus rhythm, and ejection fraction = 50% or higher and 4 to 16 weeks after CABG | Intervention I: moderate intensity continuous training for 6 weeks (3 sessions per week with 45min workouts)  Intervention II: high intensity interval training  Control: no intervention | Heart rate variability, QT interval, T wave alternans and VO2 peak | May 2020 (first enrollment) | E-mail (22/10/2022) sent to get more information about the study. However, no response. |
| “Effect of exercise trainings in patients with heart failure”[9]  (IRCT2015092916532N3) | Randomized, interventional clinical trial | Adults 40 to 70 years with heart failure and an ejection fraction of 40% undergoing coronary artery angioplasty, NYHA class I-III and stable pharmacological treatment | Intervention I: endurance training for 7 weeks (3 days a week with 45min sessions)  Intervention II: combined endurance-resistance training for 7 weeks (3 days a week with 30min endurance training sessions and sets of resistance training)  Control: no specific exercise program only conventional therapy (drug therapy, nutrition advice and recommendations on physical activity) | Heart rate variability, High-sensitivity CRP, NT-proBNP | October 2015 (first enrollment) | E-mail (22/10/2022) sent to get more information about the study and results concerning heart rate variability. However, no response. |
| “The Effect of Exercise Rehabilitation based on Different Exercise Prescriptions on the Lp-PLA2, BPV and HRV of the CHD patients after percutaneous coronary intervention”[10]  (ChiCTR2100048124) | Randomized, interventional clinical trial, parallel assignment | People with coronary heart disease after percutaneous coronary intervention | Intervention I (group B in study): exercise rehabilitation based on 6 minutes walk test  Intervention II (group C in study): exercise rehabilitation based on exercise treadmill test  Intervention III (group D in study): exercise rehabilitation based on cardio-pulmonary exercise test  Control (group A in study): standardized medication | Lipoprotein phosphorus esterase, heart rate variability, blood pressure variability, blood lipide and homocysteine | January 2019 (first enrollment) | One publication (matched to study ID) found. However, results are focused on Lipoprotein phosphorus esterase. Group A is interpreted as control group as it is the only group without training. E-mail sent (22/10/2022) to get more information about the trial and results concerning HRV. However, just received an automatic response. |
| “A prospective randomized controlled trial of Baduanjin Exercise Rehabilitation on exercise tolerance in patients with chronic ejection fraction preserved heart failure”[11]  (ChiCTR2000037742) | Randomized, interventional clinical trial, parallel assignment | Adults between 30 and 80 years with ejection fraction preserved heart failure, NYHA class I-III | Intervention: Baduanjin excercise and standard drug therapy  Control: standard drug therapy | Six minute walk test, VO_2_ peak, anaerobic threshold, oxygen uptake efficiency slope, VE/VCO_2_ slope, exercise oscillatory ventilation,maximal inspiratory pressure and heart rate variability | October 2020 (first enrollment) | E-Mail sent (22/10/2022) to get more information about the trial. No response. |
| “Exercise training in Diastolic Heart Failure - Pilot study: a prospective, randomised, controlled study to determine the effects of physical training on exercise capacity and quality of life”[12]  (ISRCTN42524037) | Randomized, interventional clinical trial, multicentre | Adults equal to or older than 45 years with at least one risk factor (diabetes, hypertension, smoking, hyperlipidaemia, overweight), written informed consent, NYHA II or III, diastolic dysfunction greater than or equal to grade one or left ventricular ejection fraction greater than or equal to 50% | Intervention: combined endurance and strength exercise training  Control: usual activity | VO_2_ peak, quality of life, early left ventricular filling-to-peak early diastolic myocardial velocity ratio, six-minute walking distance, parameter of neurohormonal acitvation, heart rate variability and turbulence, blood levels of parameters of collagen turnover, metabolic syndrome and inflammation, parameters of diastolic dysfunction, severity of diastolic dysfunction, endothelial function, safety, compliance, cardiovascular morbidity and mortality | October 2006 (first enrollment) | Results available, however, no results found concerning heart rate variability. E-Mail sent (14/11/2022) to get more information about the trial and its results concerning heart rate variability. No response |

## **Risk of bias**

**Fig S1. Risk of bias graph** illustration the results of quality assessment of the RCT with cross-over design

## **Meta-analysis^[[1]](#footnote-1)^**

Meta-analysis included 16 different studies in total [13-28].

### **Time-domain parameter**


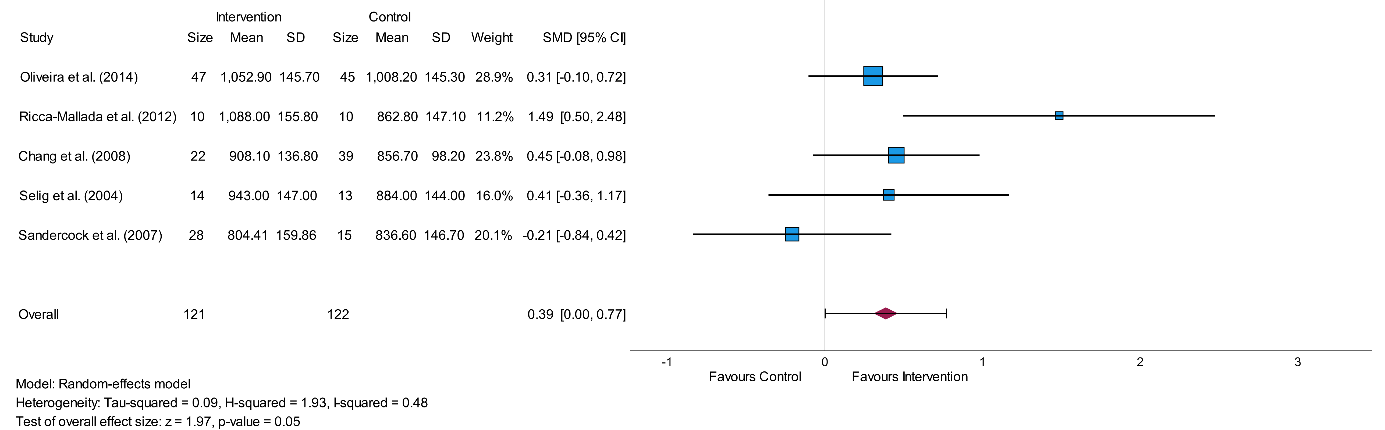


**Fig S2. Forest plot of the time-domain parameter RR-Interval for short-term assessment showing the effects of physical exercise training vs. usual care or no intervention in patients with cardiovascular diseases.** SD = standard deviation, SMD = standard mean difference, CI = confidence interval


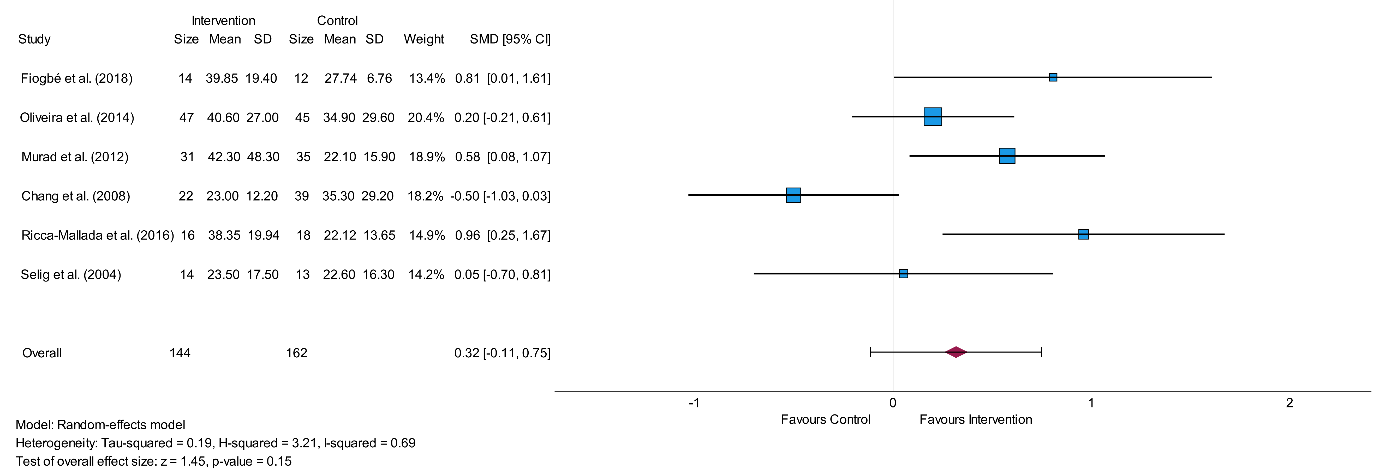


**Fig S3. Forest plot illustrating the impact of physical exercise intervention compared to usual care or no intervention on the time-domain parameter RMSSD of short-term assessment.** SD = standard deviation, SMD = standard mean difference, CI = confidence interval, RMSSD = root-mean-square difference of successive normal R-R intervals


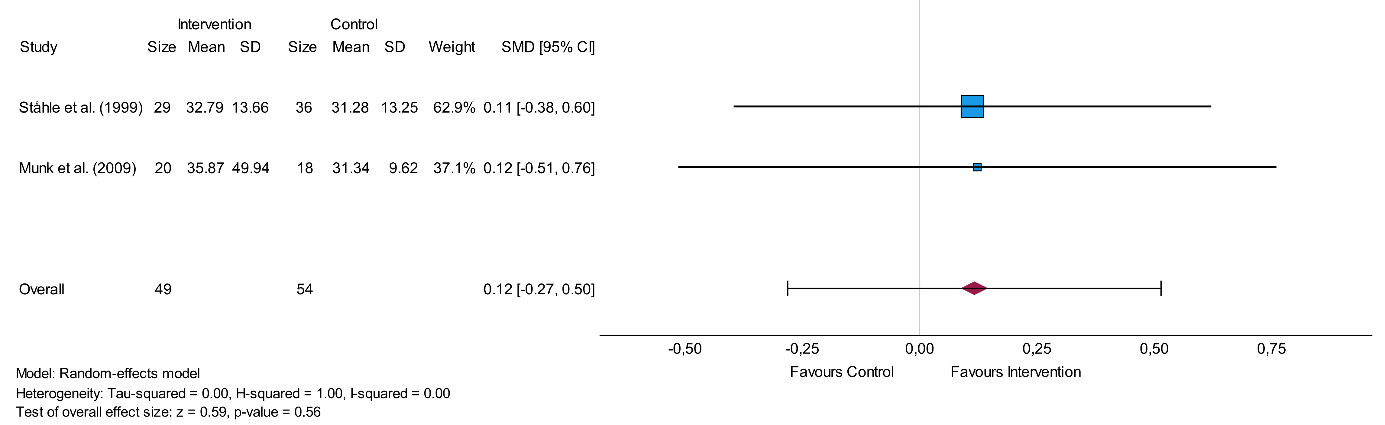


**Fig S4. Forest plot** of the time-domain parameter RMSSD for 24-hour assessment showing the effect of physical activity exercises in comparison to usual care or no intervention. SD = standard deviation, SMD = standard mean difference, CI = confidence interval, RMSSD = root-mean-square difference of successive normal R-R intervals

### **Frequency-domain parameter**


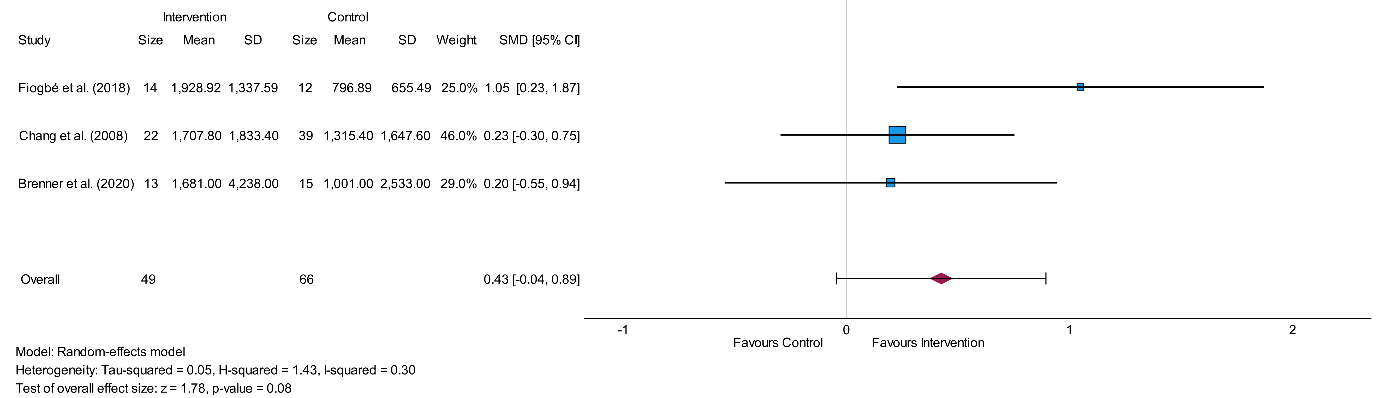


**Fig S5. Forest plot** presenting the influence of physical activity intervention vs. usual care or no intervention on the frequency-domain parameter TP of short-term assessment. SD = standard deviation, SMD = standard mean difference, CI = confidence interval, TP = total power


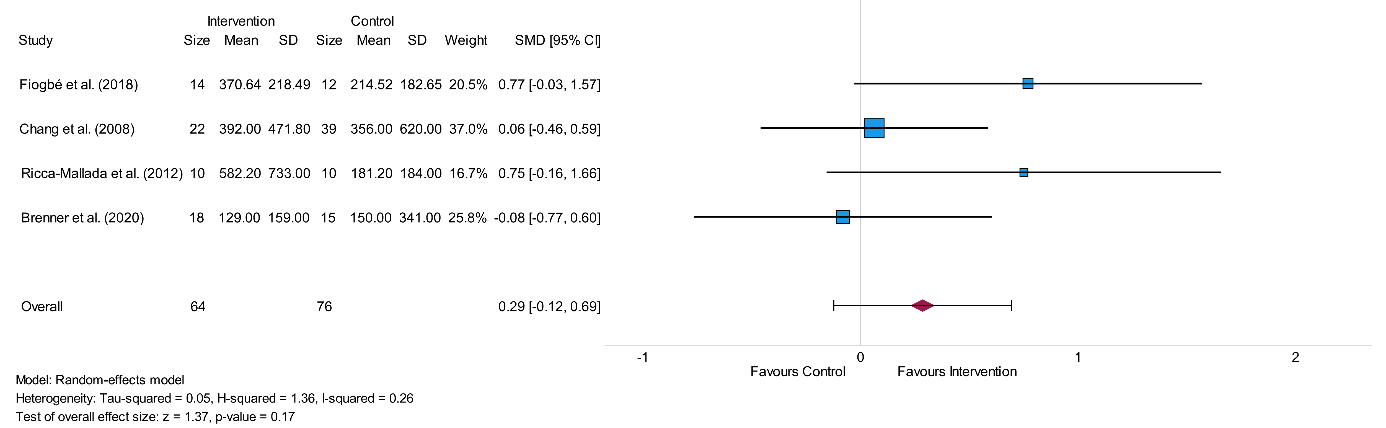


**Fig S6. Forest plot** of the frequency-domain parameter LF for short-term assessment showing the impact of physical activity exercises in comparison to the usual care or no intervention. SD = standard deviation, SMD = standard mean difference, CI = confidence interval, LF = low-frequency power


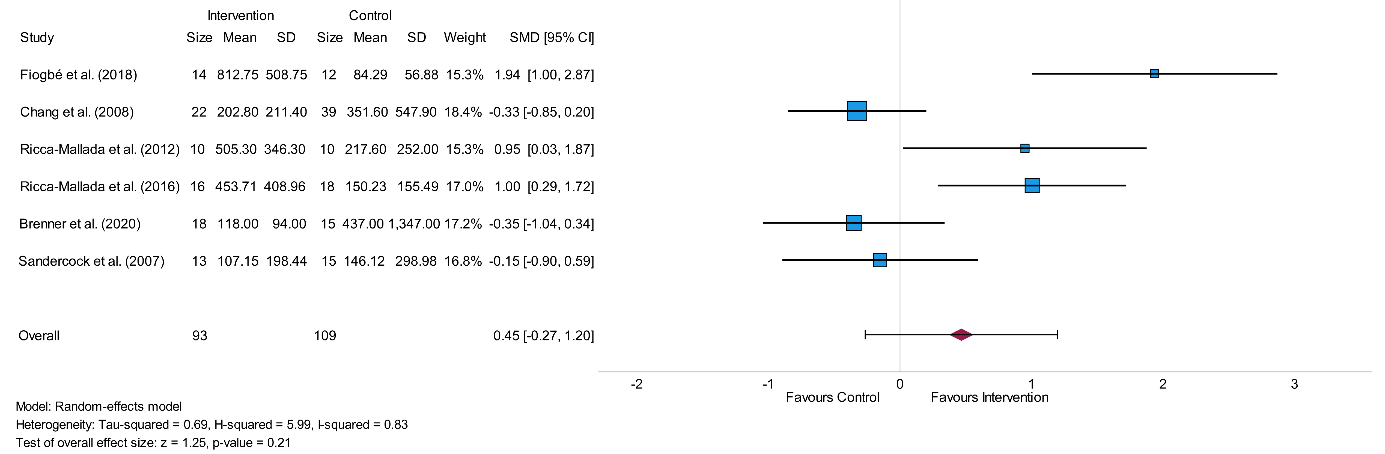


**Fig S7. Forest plot** presenting the effects of physical activity exercises on the frequency-domain parameter HF of short-term assessment compared to usual care or no intervention. SD = standard deviation, SMD = standard mean difference, CI = confidence interval, HF = high-frequency power


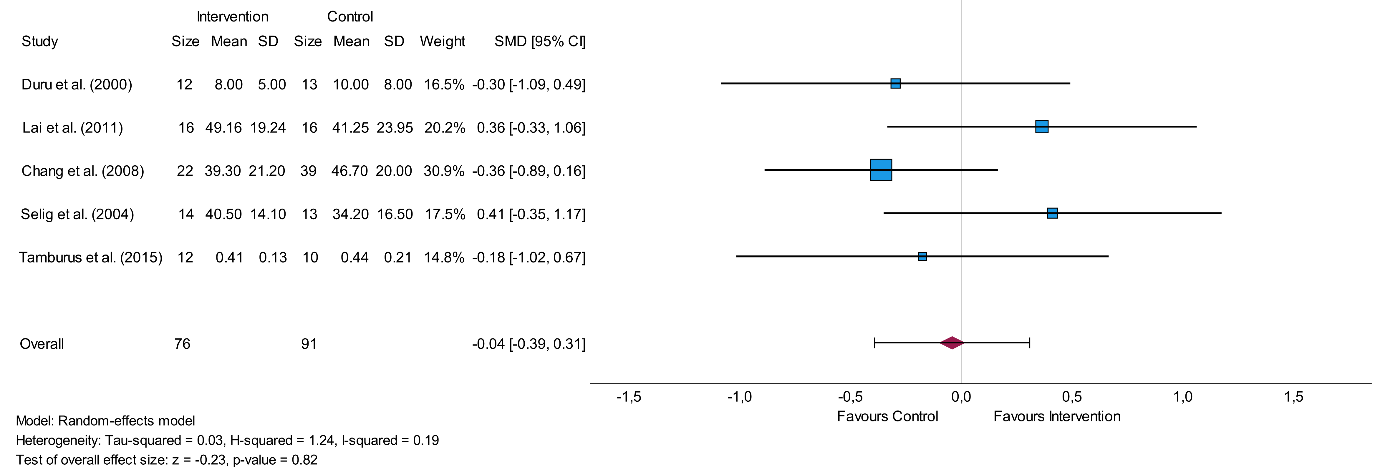


**Fig S8. Forest plot** of the frequency-domain parameter nHF for short-term assessment illustrating the impact of physical activity exercises on nHF compared to usual care or no intervention. SD = standard deviation, SMD = standard mean difference, CI = confidence interval, nHF= high-frequency power in normalized units


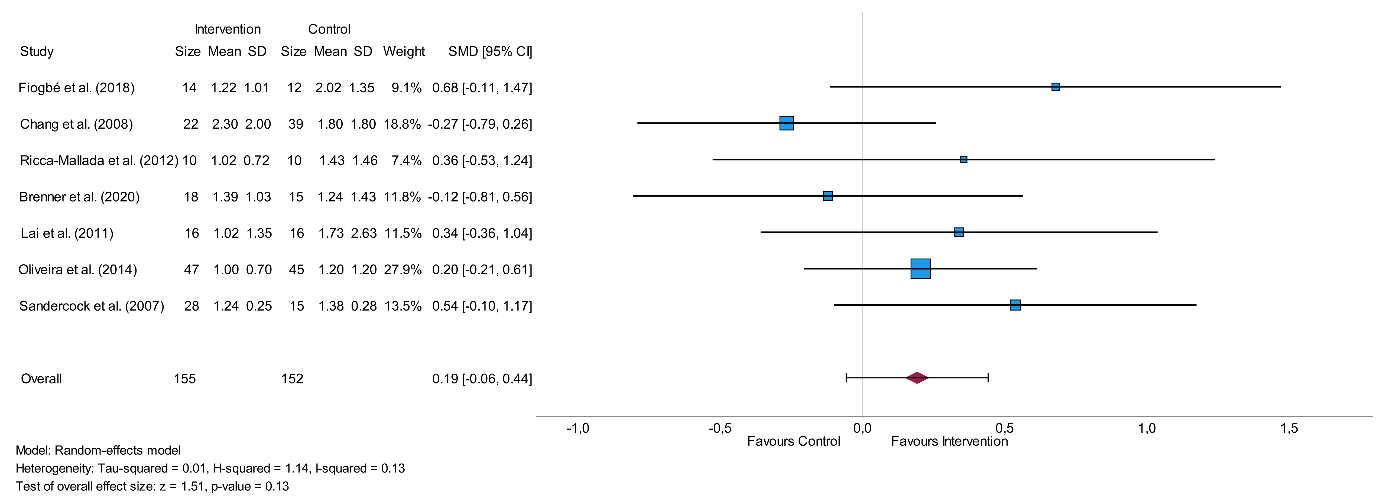


**Fig S9. Forest plot** showing the influence of physical activity exercise intervention on the frequency-domain parameter LF/HF of short-term assessment compared to usual care or no intervention. SD = standard deviation, SMD = standard mean difference, CI = confidence interval, LF/HF = ratio of low-frequency power to high-frequency power


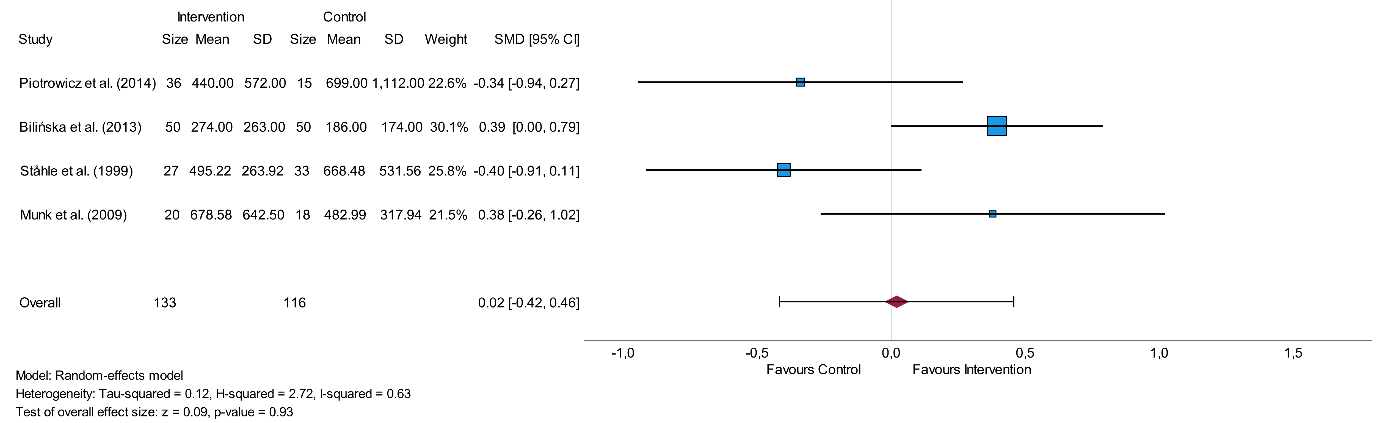


**Fig S10. Forest plot** of the frequency-domain parameter LF for 24-hour assessment showing the impact of physical activity exercises in comparison to the usual care or no intervention. SD = standard deviation, SMD = standard mean difference, CI = confidence interval, LF = low-frequency power


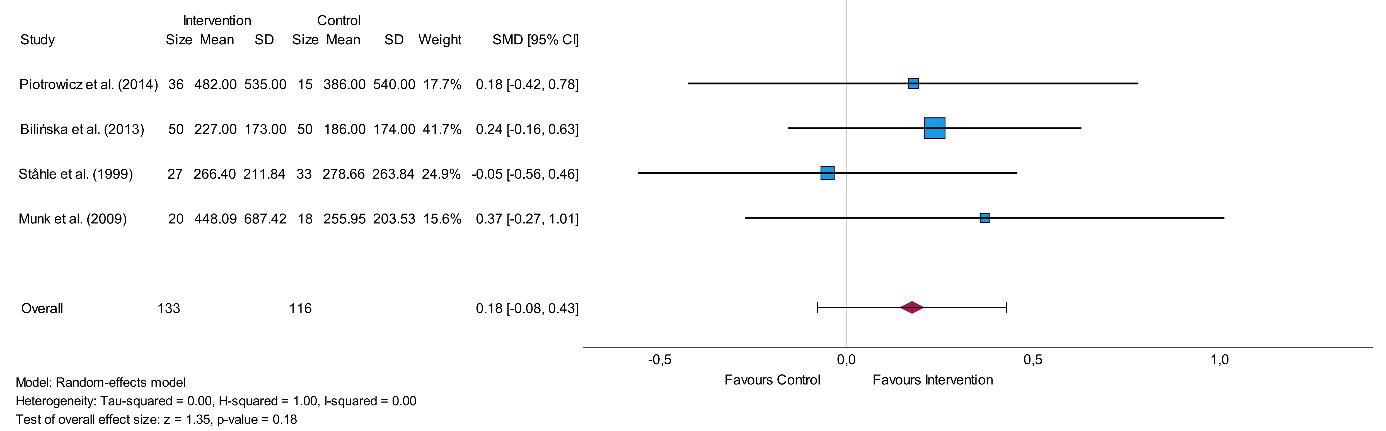


**Fig S11. Forest plot** presenting the effects of physical activity exercises on the frequency-domain parameter HF of 24-hour assessment compared to usual care or no intervention. SD = standard deviation, SMD = standard mean difference, CI = confidence interval, HF = high-frequency power


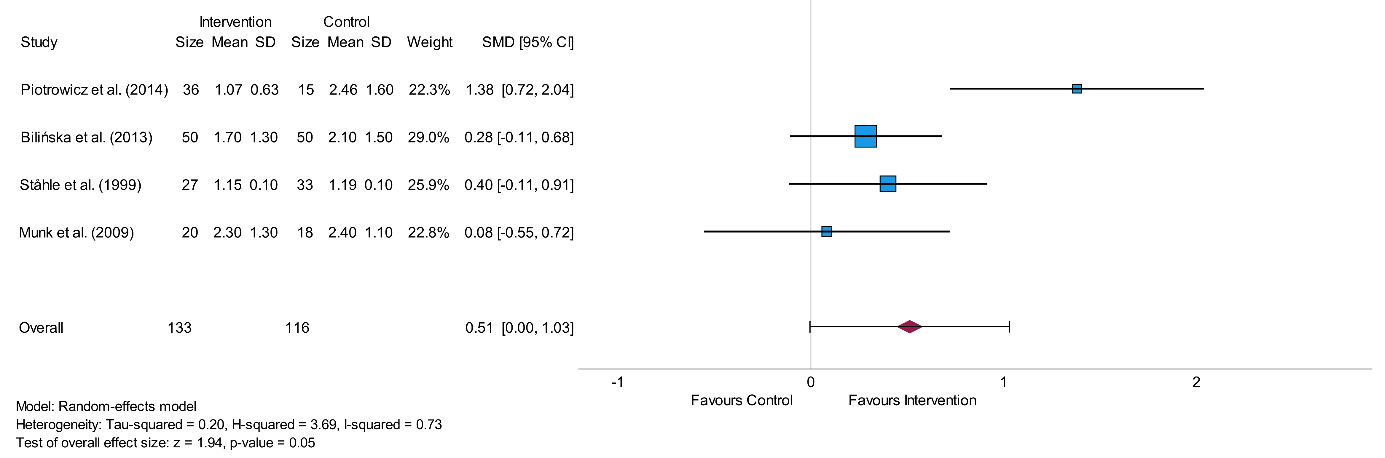


**Fig S12. Forest plot** showing the influence of physical activity exercise intervention on the frequency-domain parameter LF/HF of 24-hour assessment compared to usual care or no intervention. SD = standard deviation, SMD = standard mean difference, CI = confidence interval, LF/HF = ratio of low-frequency power to high-frequency-power

### **Subgroup analysis**

**Table S3. Results of subgroup analysis related to investigated heart diseases**

|  | **Subgroup** | **Effect Size** | **Std. Error** | **p value** |
| --- | --- | --- | --- | --- |
| **RR Interval** | myocardial infarction | 0.31 | 0.21 | 0.14 |
|  | coronary artery disease | 0.45 | 0.27 | 0.09 |
|  | congestive heart failure | 0.90 | 0.54 | 0.10 |
|  | peripheral arterial disease | -0.21 | 0.32 | 0.52 |
| **RMSSD** | myocardial infarction | 0.20 | 0.21 | 0.34 |
|  | coronary artery disease | 0.12 | 0.65 | 0.86 |
|  | congestive heart failure | 0.55 | 0.21 | 0.01 |
| **SDNN** | coronary artery disease | 0.38 | 0.42 | 0.37 |
|  | congestive heart failure | 0.44 | 0.19 | 0.02 |
|  | myocardial infarction | 0.19 | 0.19 | 0.30 |
| **TP** | coronary artery disease | 0.57 | 0.40 | 0.16 |
|  | peripheral arterial disease | 0.20 | 0.38 | 0.60 |
| **LF** | coronary artery disease | 0.35 | 0.35 | 0.31 |
|  | congestive heart failure | 0.75 | 0.46 | 0.10 |
|  | peripheral arterial disease | -0.08 | 0.35 | 0.82 |
| **HF** | coronary artery disease | 0.77 | 1.13 | 0.50 |
|  | congestive heart failure | 0.98 | 0.29 | <0.01 |
|  | peripheral arterial disease | -0.26 | 0.26 | 0.32 |

RMSSD = root-mean-square difference of successive normal R-R intervals, SDNN = standard deviation of Normal-to-Normal intervals, TP = total power, LF = low-frequency power, HF = high-frequency power

**Table S4. Results of the test regarding subgroup-homogeneity for type of disease**

|  | **Parameter** | **Number of studies** |  | **p value** |
| --- | --- | --- | --- | --- |
| **Short--term** | RR Interval | 5 |  | 0.26 |
|  | RMSSD | 6 |  | 0.46 |
|  | SDNN | 7 |  | 0.64 |
|  | TP | 3 |  | 0.50 |
|  | LF | 4 |  | 0.34 |
|  | HF | 6 |  | 0.01 |

RMSSD = root-mean-square difference of successive normal R-R intervals, SDNN = standard deviation of Normal-to-Normal intervals, TP = total power, LF = low-frequency power, HF = high-frequency power

**Table S5. Results of subgroup analysis concerning intervention type**

|  | **Subgroup** | **Effect Size** | **Std. Error** | **p value** |
| --- | --- | --- | --- | --- |
| **RR Interval** | aerobic exercise training | 0.31 | 0.21 | 0.14 |
|  | resistance exercise training | 0.41 | 0.39 | 0.30 |
|  | aerobic and resistance training | 1.49 | 0.51 | <0.01 |
|  | T'ai Chi training | 0.45 | 0.27 | 0.09 |
|  | walking exercise | -0.21 | 0.32 | 0.52 |
| **RMSSD** | aerobic exercise training | 0.51 | 0.21 | 0.01 |
|  | resistance exercise training | 0.05 | 0.39 | 0.89 |
|  | T'ai Chi training | -0.50 | 0.27 | 0.06 |
|  | water aerobic exercise training | 0.81 | 0.41 | 0.05 |
| **SDNN** | aerobic exercise training | 0.32 | 0.18 | 0.07 |
|  | aerobic and resistance training | 0.70 | 0.46 | 0.13 |
|  | walking exercise | 0.84 | 0.37 | 0.02 |
|  | resistance exercise training | 0.05 | 0.39 | 0.89 |
|  | T'ai Chi training | -0.01 | 0.27 | 0.99 |
|  | walking and cycling exercises | 0.27 | 0.40 | 0.50 |
| **TP** | T'ai Chi training | 0.23 | 0.27 | 0.39 |
|  | walking exercise | 0.20 | 0.38 | 0.60 |
|  | water aerobic exercise training | 1.05 | 0.42 | 0.01 |
| **LF** | aerobic and resistance training | 0.75 | 0.46 | 0.10 |
|  | T'ai Chi training | 0.06 | 0.27 | 0.81 |
|  | walking exercise | -0.08 | 0.35 | 0.82 |
|  | water aerobic exercise training | 0.77 | 0.41 | 0.06 |
| **HF** | aerobic exercise training | 1.00 | 0.36 | 0.01 |
|  | aerobic and resistance training | 0.95 | 0.47 | 0.04 |
|  | T'ai Chi training | -0.33 | 0.27 | 0.23 |
|  | walking exercise | -0.26 | 0.26 | 0.32 |
|  | water aerobic exercise training | 1.94 | 0.48 | <0.01 |

RMSSD = root-mean-square difference of successive normal R-R intervals, SDNN = standard deviation of Normal-to-Normal intervals, TP = total power, LF = low-frequency power, HF = high-frequency power

**Table S6. Results of the test regarding subgroup-homogeneity for intervention type**

|  | **Parameter** | **Number of studies** |  | **p value** |
| --- | --- | --- | --- | --- |
| **Short-term** | RR Interval | 5 |  | 0.08 |
|  | RMSSD | 6 |  | 0.01 |
|  | SDNN | 7 |  | 0.46 |
|  | TP | 3 |  | 0.21 |
|  | LF | 4 |  | 0.24 |
|  | HF | 6 |  | <0.001 |

RMSSD = root-mean-square difference of successive normal R-R intervals, SDNN = standard deviation of Normal-to-Normal intervals, TP = total power, LF = low-frequency power, HF = high-frequency power

**Table S7. Results of subgroup analysis related to study design**

|  | **Subgroup** | **Effect Size** | **Std. Error** | **p value** |
| --- | --- | --- | --- | --- |
| **RR Int.** | Non-RCT | 0.45 | 0.27 | 0.09 |
|  | RCT | 0.41 | 0.30 | 0.17 |
| **RMSSD** | Non-RCT | -0.50 | 0.27 | 0.06 |
|  | RCT | 0.47 | 0.16 | <0.01 |
| **SDNN** | Non-RCT | 0.38 | 0.42 | 0.37 |
|  | RCT | 0.31 | 0.13 | 0.02 |
| **TP** | Non-RCT | 0.23 | 0.27 | 0.39 |
|  | RCT | 0.60 | 0.42 | 0.15 |
| **LF** | Non-RCT | 0.06 | 0.27 | 0.81 |
|  | RCT | 0.43 | 0.30 | 0.15 |
| **HF** | Non-RCT | -0.33 | 0.27 | 0.23 |
|  | RCT | 0.65 | 0.42 | 0.12 |

RR Int. = RR-Interval, RMSSD = root-mean-square difference of successive normal R-R intervals, SDNN = standard deviation of Normal-to-Normal intervals, TP = total power, LF = low-frequency power, HF = high-frequency power, RCT = randomized controlled trial

**Table S8. Results of the test regarding subgroup-homogeneity for study design**

|  | **Parameter** | **Number of studies** |  | **p value** |
| --- | --- | --- | --- | --- |
| **Short-term** | RR Interval | 5 |  | 0.91 |
|  | RMSSD | 6 |  | 0.002 |
|  | SDNN | 7 |  | 0.88 |
|  | TP | 3 |  | 0.45 |
|  | LF | 4 |  | 0.36 |
|  | HF | 6 |  | 0.05 |

RMSSD = root-mean-square difference of successive normal R-R intervals, SDNN = standard deviation of Normal-to-Normal intervals, TP = total power, LF = low-frequency power, HF = high-frequency power

**Table S9. Results of sensitivity analysis with mean difference as effect size**

|  | **Parameter** | **Mean Difference** | **Std. Error** | **p value** |
| --- | --- | --- | --- | --- |
| **short-term** | RR Interval [ms] | 58.55 | 31.60 | 0.06 |
|  | RMSSD [ms] | 8.09 | 5.55 | 0.14 |
|  | SDNN [ms] | 6.63 | 2.13 | <0.01 |
|  | TP [ms^2^] | 781.75 | 369.30 | 0.03 |
|  | LF [ms^2^] | 130.28 | 70.70 | 0.07 |
|  | LF [ms^2^/Hz] | 131.94 | 202.86 | 0.52 |
|  | HF [ms^2^] | 285.81 | 438.61 | 0.51 |
|  | HF [ms^2^/Hz] | 258.71 | 79.76 | <0.01 |
| **24-hour** | RMSSD [ms] | 1.73 | 3.23 | 0.59 |
|  | SDNN [ms] | 6.78 | 5.15 | 0.19 |
|  | LF [ms^2^] | -9.76 | 183.23 | 0.96 |
|  | LF [ms^2^/Hz] | -8.48 | 155.47 | 0.96 |
|  | HF [ms^2^] | 30.13 | 82.87 | 0.72 |
|  | HF [ms^2^/Hz] | 43.33 | 33.96 | 0.20 |

RMSSD = root-mean-square difference of successive normal R-R intervals, SDNN = standard deviation of Normal-to-Normal intervals, TP = total power, LF = low-frequency power, HF = high-frequency power

# **References**

[1] Clark JM, Sanders S, Carter M, Honeyman D, Cleo G, Auld Y, et al. Improving the translation of search strategies using the Polyglot Search Translator: a randomized controlled trial. J Med Libr Assoc. 2020;108(2):195-207. Epub 20200401. doi: 10.5195/jmla.2020.834. PubMed PMID: 32256231; PubMed Central PMCID: PMCPMC7069833.

[2] NCT00259948. Aerobic Exercise Training & the Autonomic System In Patients After Myocardial Infarction or Stroke 2018 [updated 2018/05/31]. Available from: <https://clinicaltrials.gov/show/NCT00259948>.

[3] NCT04981197. Effects of Baduanjin Exercise on Heart Failure Patients [Clinical trial registration]. clinicaltrials.gov; 2021 [updated July 18, 2021]. Available from: <https://clinicaltrials.gov/ct2/show/NCT04981197?term=NCT04981197&draw=2&rank=1>.

[4] NCT00194701. Exercise to Improve Sleep in Heart Failure [Clinical trial registration]. clinicaltrials.gov; 2008 [updated January 2, 2008]. Available from: <https://clinicaltrials.gov/ct2/show/NCT00194701?term=NCT00194701&draw=2&rank=1>.

[5] NCT00012883. Home Walking Exercise Training in Advanced Heart Failure 2018 [updated 2018/05/31]. Available from: <https://clinicaltrials.gov/show/NCT00012883>.

[6] NCT04333615. Self Selected Exercise Intensity in PAD Patients 2020 [updated 2020/04/30]. Available from: <https://clinicaltrials.gov/show/NCT04333615>.

[7] RBR-3y8w4w3. Promotion of Physical Activity in primary health care 2021 [updated 2021/05/31]. Available from: <https://trialsearch.who.int/Trial2.aspx?TrialID=RBR-3y8w4w3>.

[8] IRCT20220326054352N1. Comparison of high and moderate intensity cardiac rehabilitation after CABG 2022 [updated 2022/06/30]. Available from: <https://trialsearch.who.int/Trial2.aspx?TrialID=IRCT20220326054352N1>.

[9] IRCT2015092916532N3. Effect of exercise trainings in patients with heart failure 2019 [updated 2019/03/31]. Available from: <https://trialsearch.who.int/Trial2.aspx?TrialID=IRCT2015092916532N3>.

[10] ChiCTR2100048124. The Effect of Exercise Rehabilitation based on Different Exercise Prescriptions on the Lp-PLA2, BPV and HRV of the CHD patients after percutaneous coronary intervention 2022 [updated 2022/08/31]. Available from: <https://trialsearch.who.int/Trial2.aspx?TrialID=ChiCTR2100048124>.

[11] ChiCTR2000037742. A prospective randomized controlled trial of Baduanjin Exercise Rehabilitation on exercise tolerance in patients with chronic ejection fraction preserved heart failure 2020. Available from: <https://trialsearch.who.int/Trial2.aspx?TrialID=ChiCTR2000037742>.

[12] ISRCTN42524037. Exercise training in Diastolic Heart Failure - Pilot study: a prospective, randomised, controlled study to determine the effects of physical training on exercise capacity and quality of life 2019 [updated 2019/03/31]. Available from: <https://trialsearch.who.int/Trial2.aspx?TrialID=ISRCTN42524037>.

[13] Oliveira NL, Ribeiro F, Teixeira M, Campos L, Alves AJ, Silva G, et al. Effect of 8-week exercise-based cardiac rehabilitation on cardiac autonomic function: A randomized controlled trial in myocardial infarction patients. Am Heart J. 2014;167(5):753-61 e3. Epub 20140217. doi: 10.1016/j.ahj.2014.02.001. PubMed PMID: 24766987.

[14] Ricca-Mallada R, Migliaro ER, Piskorski J, Guzik P. Exercise training slows down heart rate and improves deceleration and acceleration capacity in patients with heart failure. Journal of electrocardiology. 2012;45(3):214-9. doi: 10.1016/j.jelectrocard.2012.01.002.

[15] Chang R-Y, Koo M, Yu Z-R, Kan C-B, Chu I-T, Hsu C-T, et al. The effect of t'ai chi exercise on autonomic nervous function of patients with coronary artery disease. Journal of alternative and complementary medicine (New York, NY). 2008;14(9):1107-13. doi: 10.1089/acm.2008.0166.

[16] Selig SE, Carey MF, Menzies DG, Patterson J, Geerling RH, Williams AD, et al. Moderate-intensity resistance exercise training in patients with chronic heart failure improves strength, endurance, heart rate variability, and forearm blood flow. Journal of cardiac failure. 2004;10(1):21-30. doi: 10.1016/s1071-9164(03)00583-9.

[17] Sandercock GR, Hodges LD, Das SK, Brodie DA. The impact of short term supervised and home-based walking programmes on heart rate variability in patients with peripheral arterial disease. J Sports Sci Med. 2007;6(4):471-6. Epub 20071201. PubMed PMID: 24149480; PubMed Central PMCID: PMCPMC3794487.

[18] Fiogbé E, Ferreira R, Sindorf MAG, Tavares SA, de Souza KP, de Castro Cesar M, et al. Water exercise in coronary artery disease patients, effects on heart rate variability, and body composition: A randomized controlled trial. Physiotherapy research international : the journal for researchers and clinicians in physical therapy. 2018;23(3):e1713. doi: 10.1002/pri.1713.

[19] Murad K, Brubaker PH, Fitzgerald DM, Morgan TM, Goff DCJ, Soliman EZ, et al. Exercise training improves heart rate variability in older patients with heart failure: a randomized, controlled, single-blinded trial. Congestive heart failure (Greenwich, Conn). 2012;18(4):192-7. doi: 10.1111/j.1751-7133.2011.00282.x.

[20] Ricca-Mallada R, Migliaro ER, Silvera G, Chiappella L, Frattini R, Ferrando-Castagnetto F. Functional outcome in chronic heart failure after exercise training: Possible predictive value of heart rate variability. Annals of physical and rehabilitation medicine. 2016;60(2):87-94. doi: 10.1016/j.rehab.2016.12.003.

[21] Stâhle A, Nordlander R, Bergfeldt L. Aerobic group training improves exercise capacity and heart rate variability in elderly patients with a recent coronary event. A randomized controlled study. European heart journal. 1999;20(22):1638-46. doi: 10.1053/euhj.1999.1715.

[22] Munk PS, Butt N, Larsen AI. High-intensity interval exercise training improves heart rate variability in patients following percutaneous coronary intervention for angina pectoris. International journal of cardiology. 2010;145(2):312-4. doi: 10.1016/j.ijcard.2009.11.015.

[23] Brenner IKM, Brown CA, Hains SJM, Tranmer J, Zelt DT, Brown PM. Low-Intensity Exercise Training Increases Heart Rate Variability in Patients With Peripheral Artery Disease. Biological research for nursing. 2020;22(1):24-33. doi: 10.1177/1099800419884642.

[24] Tamburus NY, Paula RFL, Kunz VC, César MC, Moreno MA, da Silva E. Interval training based on ventilatory anaerobic threshold increases cardiac vagal modulation and decreases high-sensitivity c-reative protein: randomized clinical trial in coronary artery disease. Brazilian journal of physical therapy. 2015;19(6):441-50. doi: 10.1590/bjpt-rbf.2014.0124.

[25] Lai F-C, Tu S-T, Huang C-H, Jeng C. A home-based exercise program improves heart rate variability and functional capacity among postmenopausal women with coronary artery disease. The Journal of cardiovascular nursing. 2011;26(2):137-44. doi: 10.1097/JCN.0b013e3181ed9424.

[26] Duru F, Candinas R, Dziekan G, Goebbels U, Myers J, Dubach P. Effect of exercise training on heart rate variability in patients with new-onset left ventricular dysfunction after myocardial infarction. American heart journal. 2000;140(1):157-61. doi: 10.1067/mhj.2000.106606.

[27] Bilińska M, Kosydar-Piechna M, Mikulski T, Piotrowicz E, Gasiorowska A, Piotrowski W, et al. Influence of aerobic training on neurohormonal and hemodynamic responses to head-up tilt test and on autonomic nervous activity at rest and after exercise in patients after bypass surgery. Cardiology Journal. 2013;20(1):17-24. doi: 10.5603/CJ.2013.0004.

[28] Piotrowicz E, Piotrowski W, Piotrowicz R. Influence of home-based telemonitored Nordic walking training on autonomic nervous system balance in heart failure patients Arch Med Sci. 2014;11(6):1205-12. Epub 20151211. doi: 10.5114/aoms.2015.56346. PubMed Central PMCID: PMCPMC4697054.

1. Please use the zoom function to increase the forest plots [↑](#footnote-ref-1)
