# Supplementary figures and images for: Beneficial impacts of physical activity on heart rate variability: A systematic review and meta-analysis
Source: PLoS One. 2024 Apr 5;19(4):e0299793. doi: 10.1371/journal.pone.0299793 (PMC10997132; doi:10.1371/journal.pone.0299793)

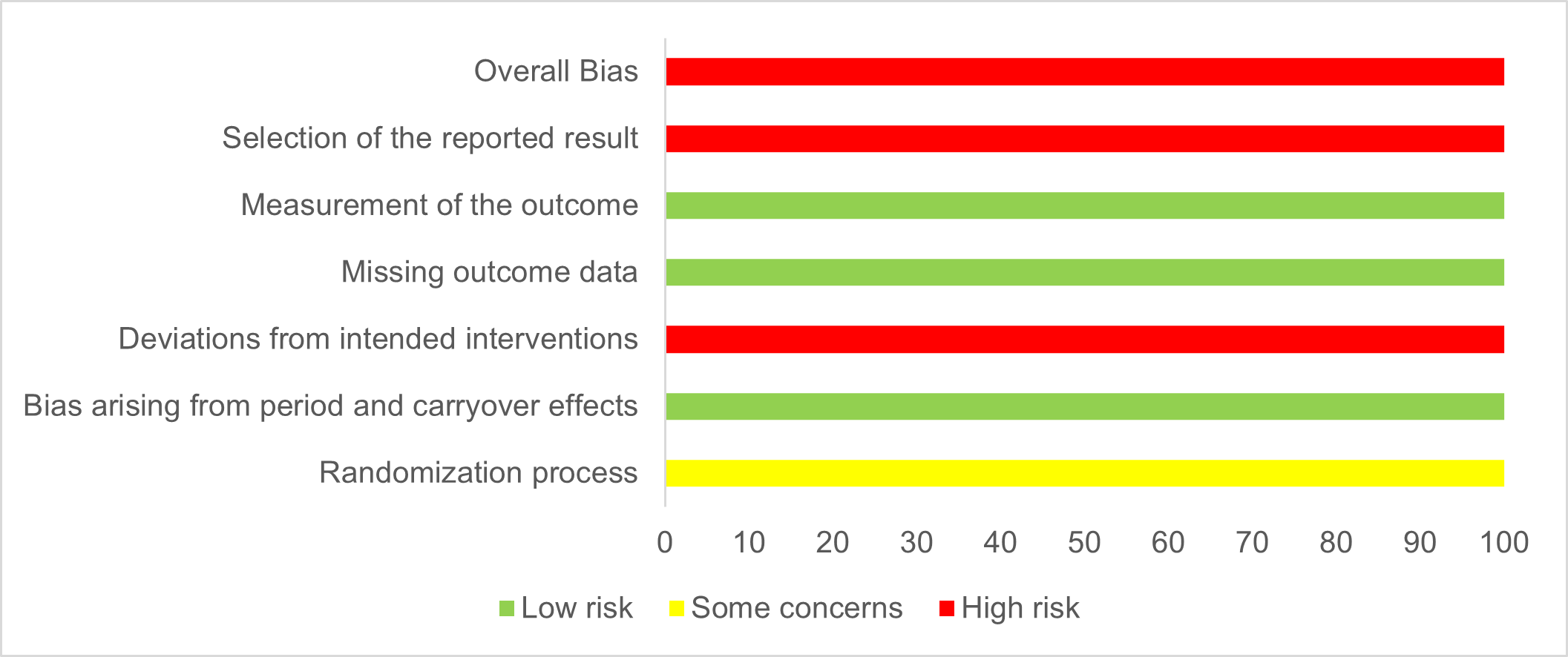

Supplement: S1 Fig — (TIF) [file pone.0299793.s002.tif]

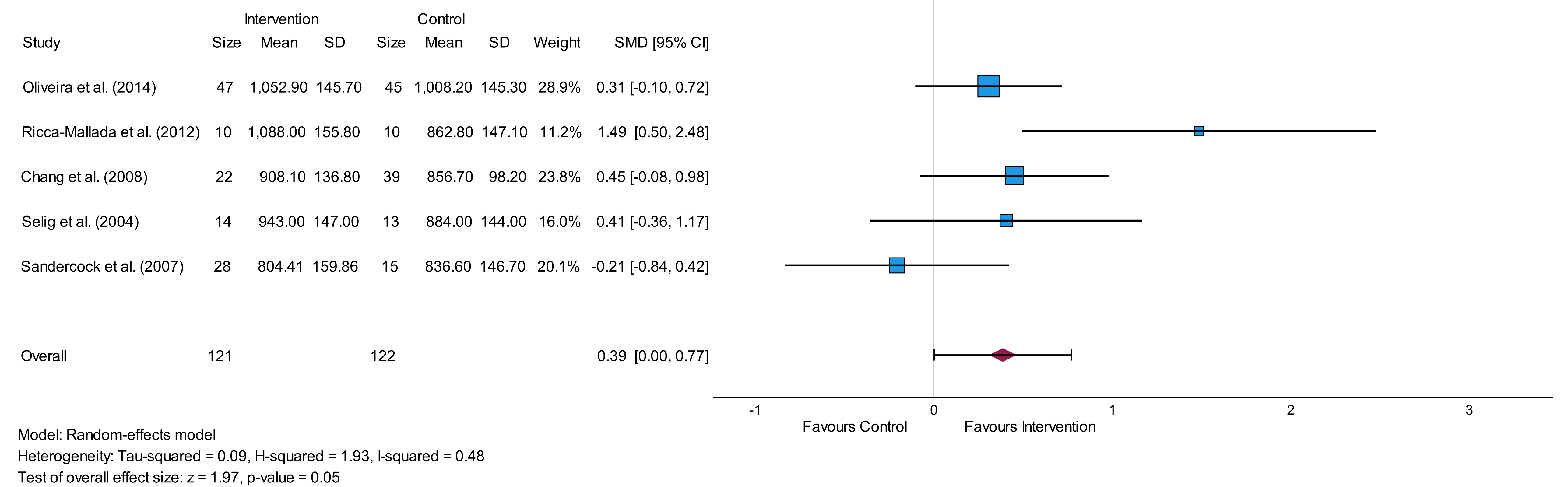

Supplement: S2 Fig — (TIFF) [file pone.0299793.s003.tiff]

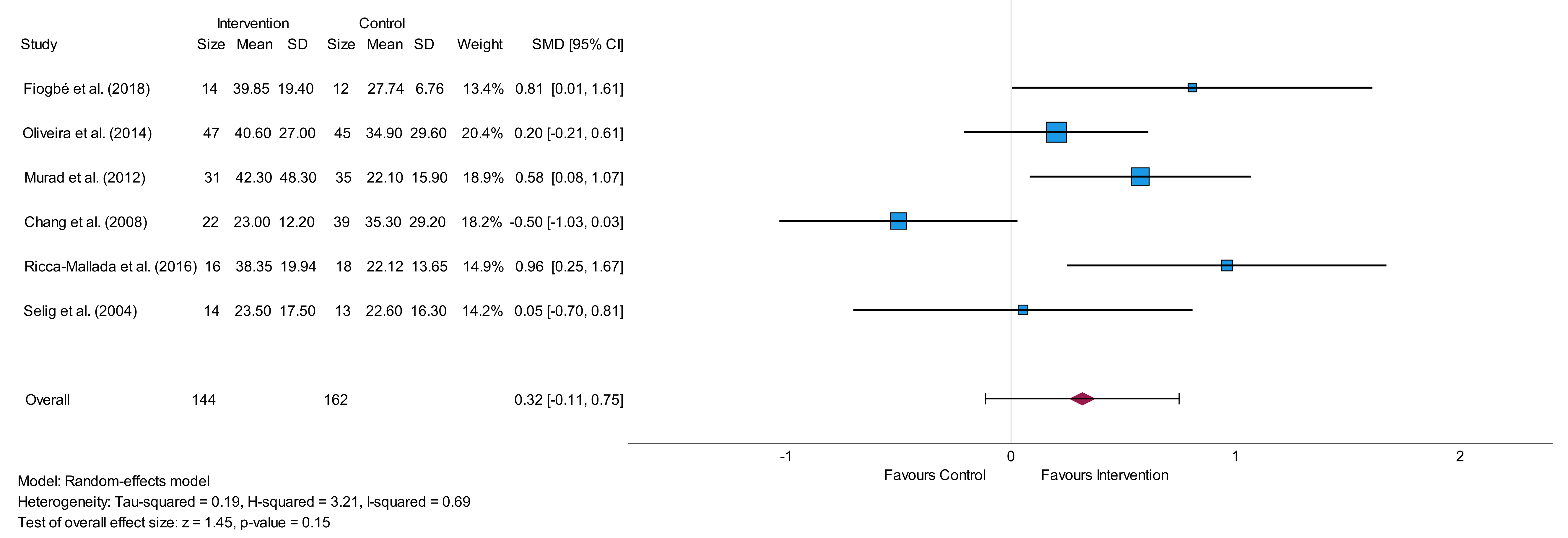

Supplement: S3 Fig — (TIFF) [file pone.0299793.s004.tiff]

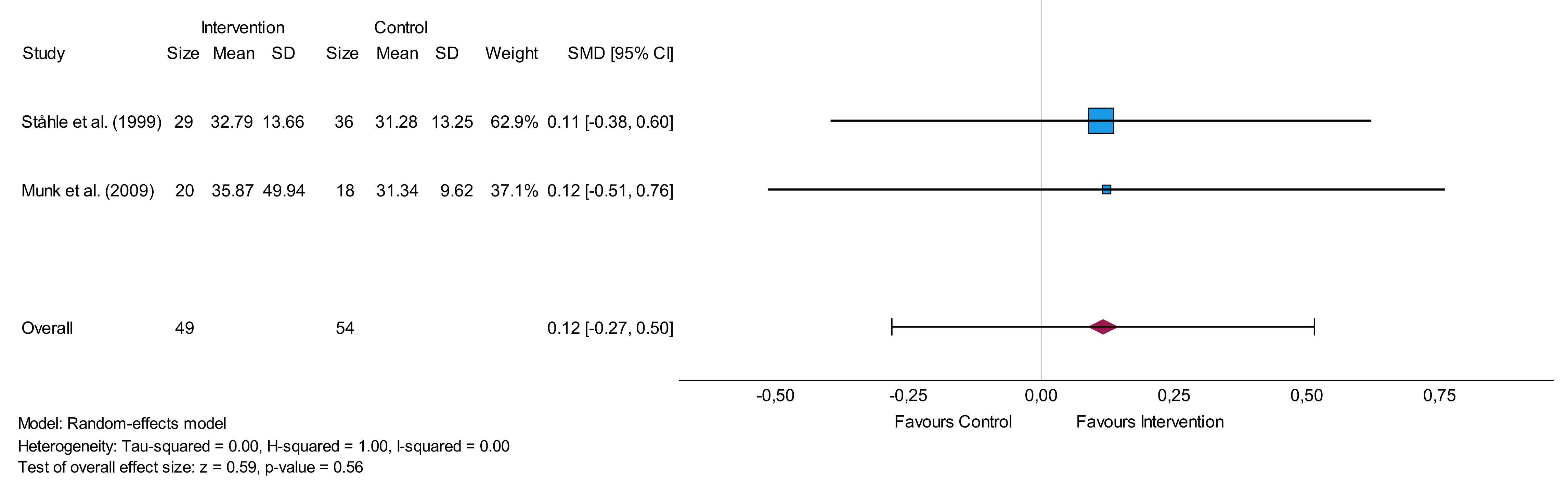

Supplement: S4 Fig — (TIFF) [file pone.0299793.s005.tiff]

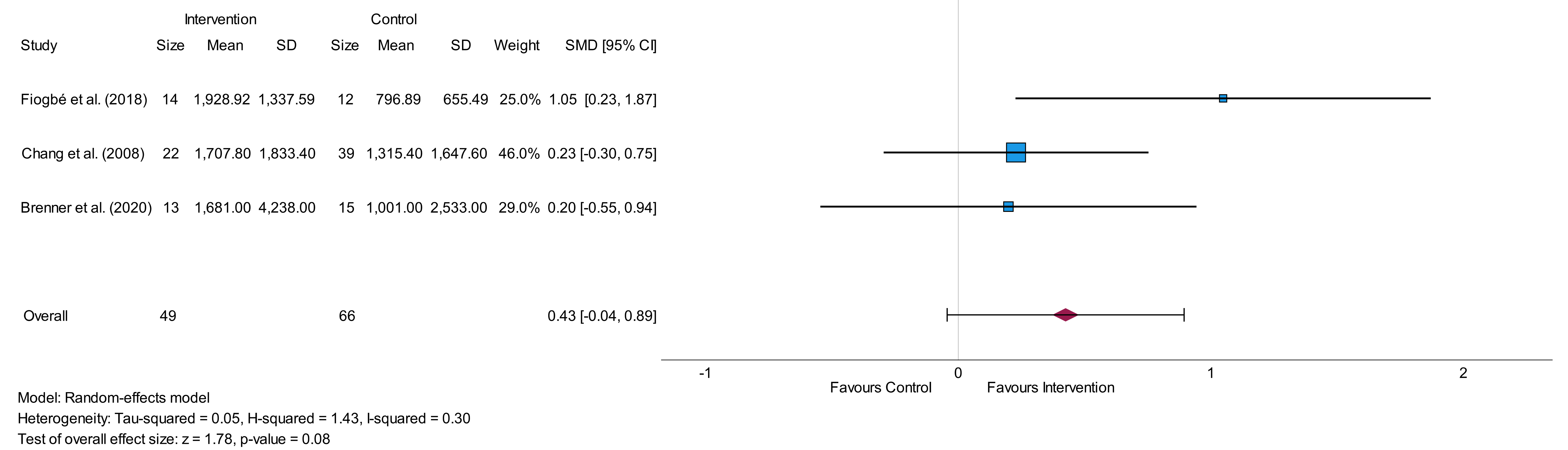

Supplement: S5 Fig — (TIFF) [file pone.0299793.s006.tiff]

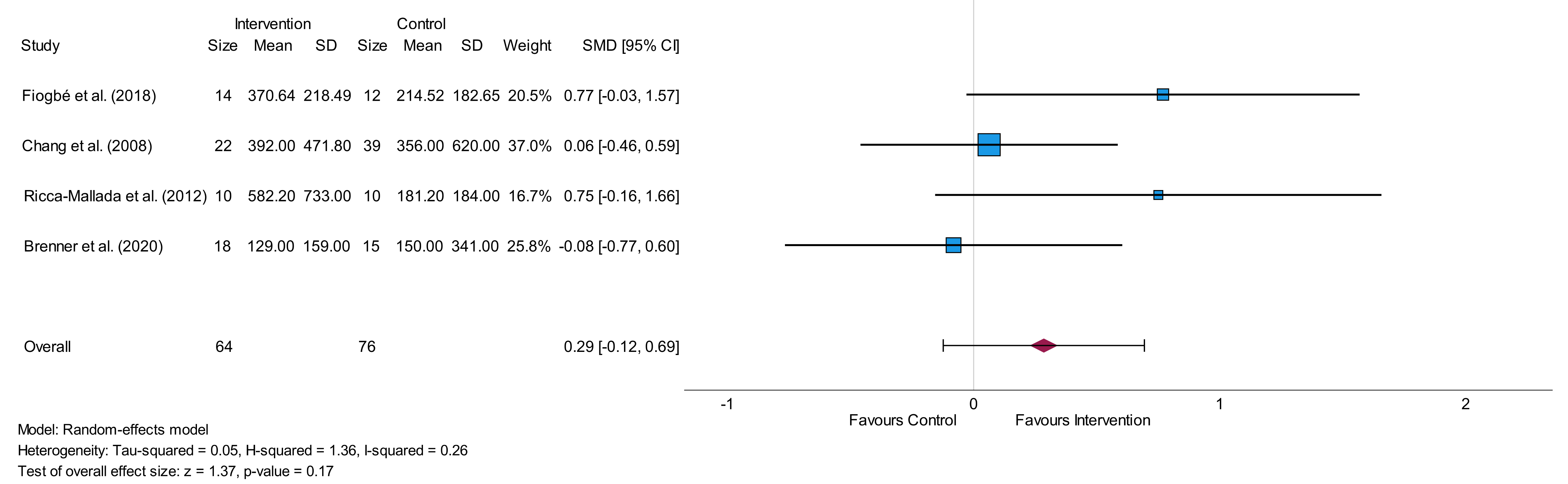

Supplement: S6 Fig — (TIFF) [file pone.0299793.s007.tiff]

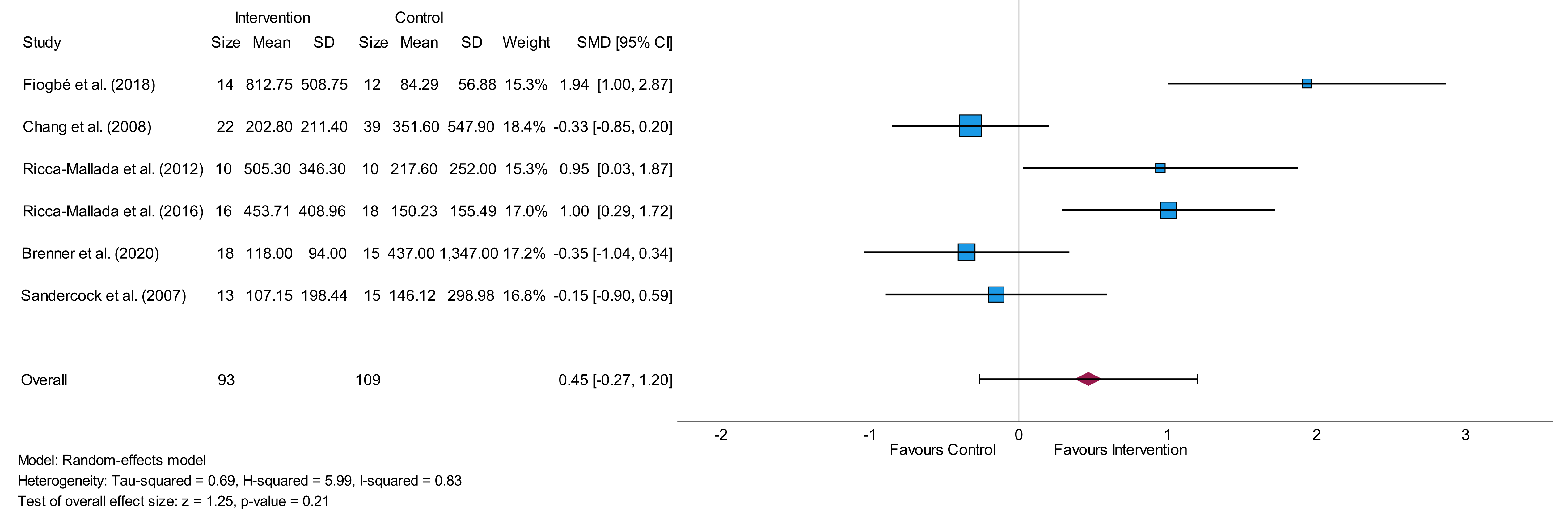

Supplement: S7 Fig — (TIFF) [file pone.0299793.s008.tiff]

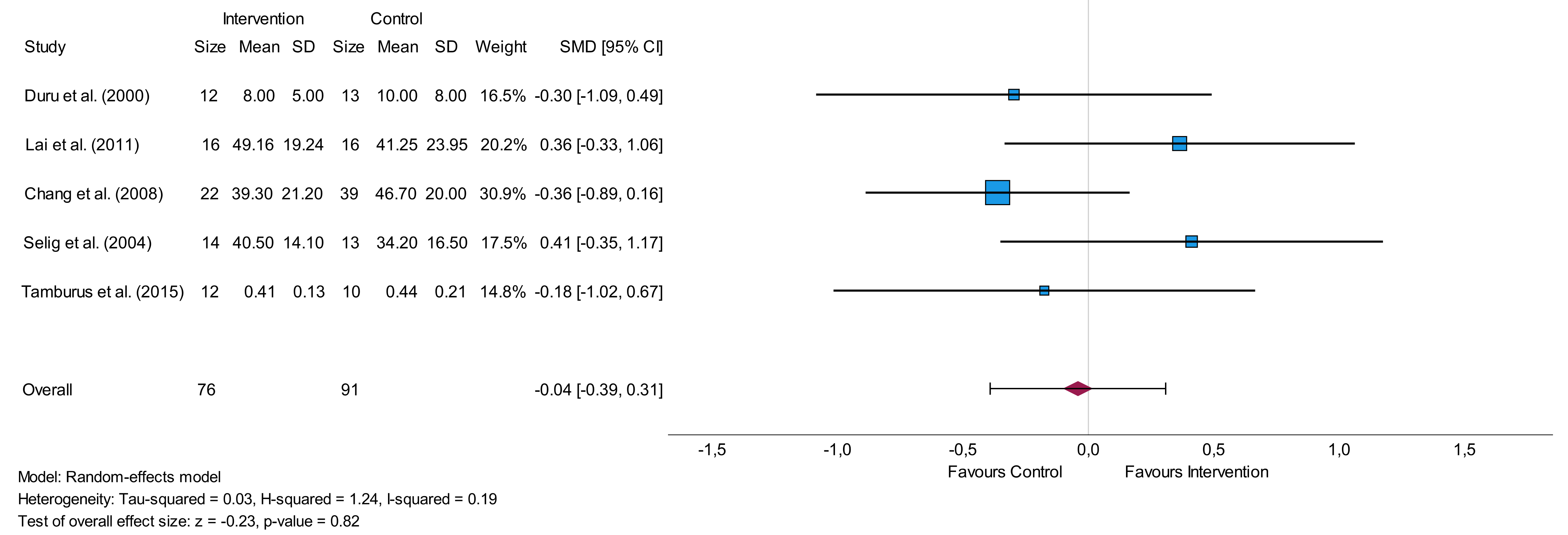

Supplement: S8 Fig — (TIFF) [file pone.0299793.s009.tiff]

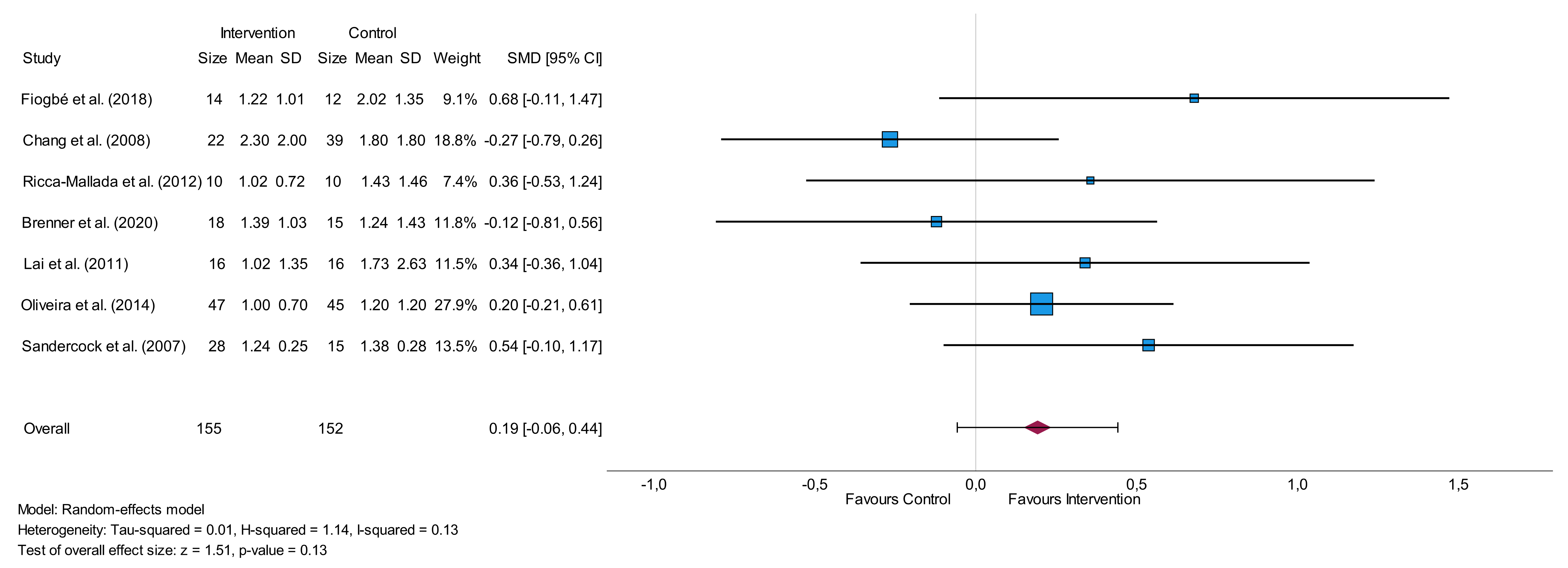

Supplement: S9 Fig — (TIFF) [file pone.0299793.s010.tiff]

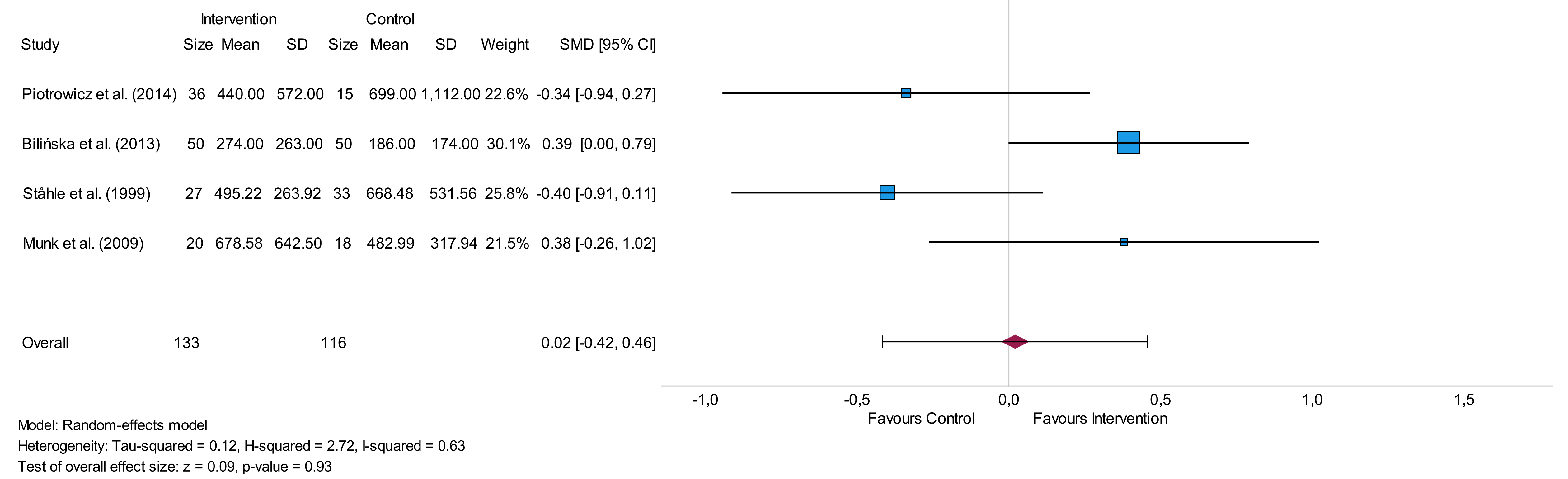

Supplement: S10 Fig — (TIFF) [file pone.0299793.s011.tiff]

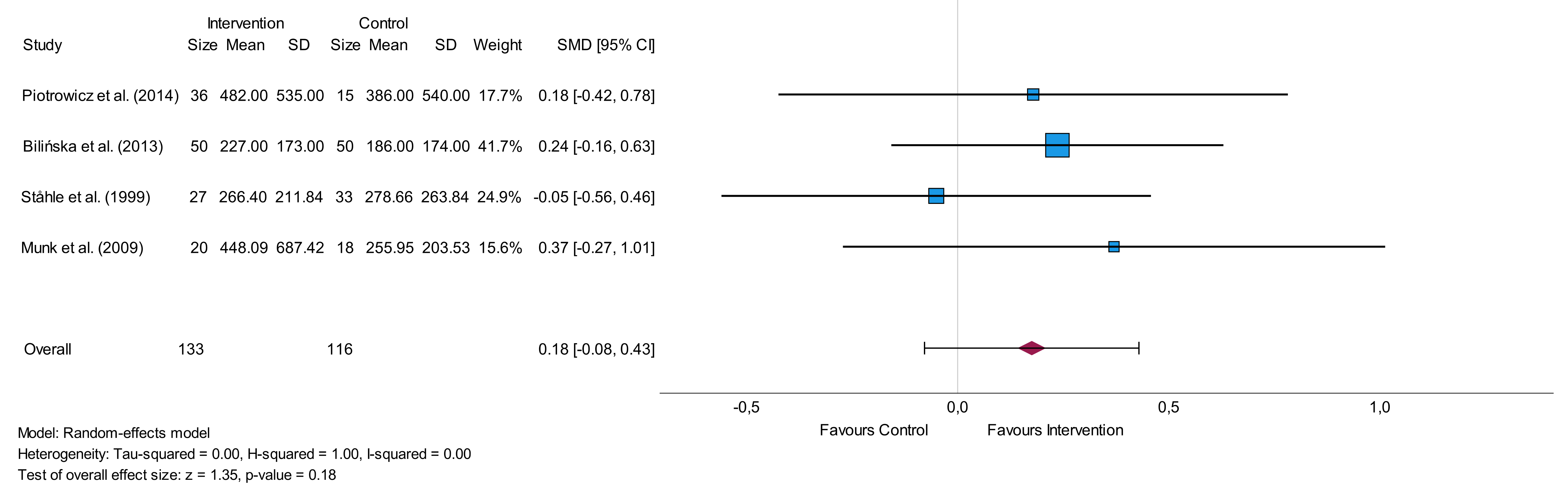

Supplement: S11 Fig — (TIFF) [file pone.0299793.s012.tiff]

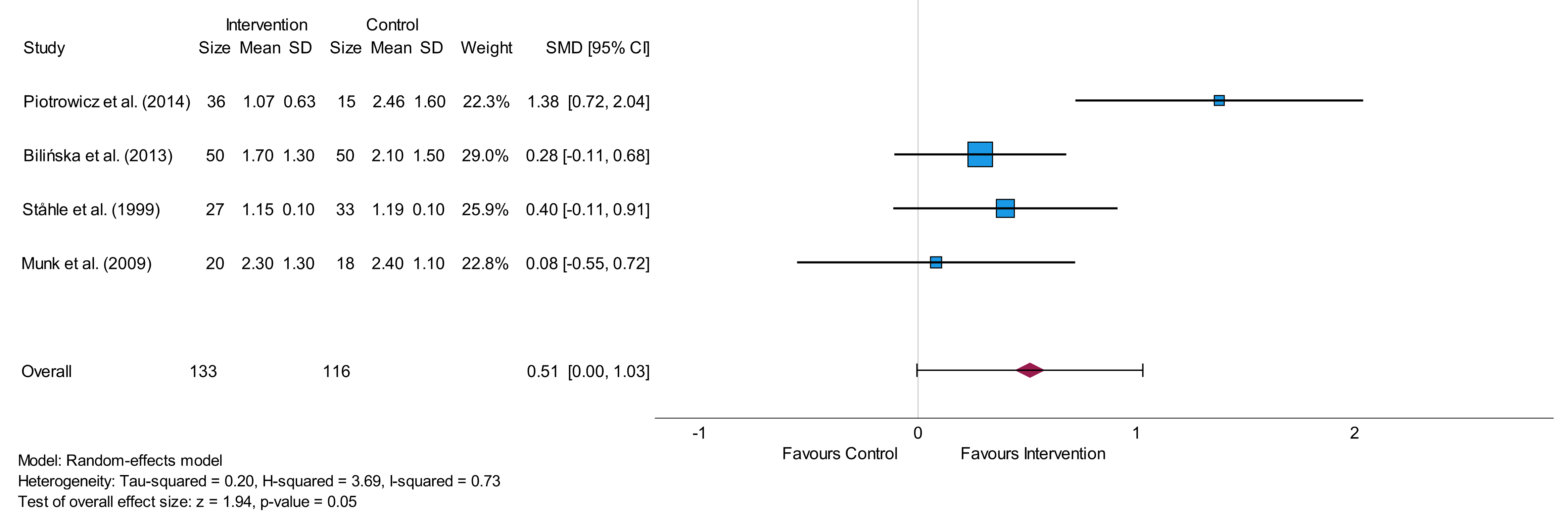

Supplement: S12 Fig — (TIFF) [file pone.0299793.s013.tiff]
